# Placental Abnormalities are Associated With Specific Windows of Embryo Culture in a Mouse Model

**DOI:** 10.3389/fcell.2022.884088

**Published:** 2022-04-25

**Authors:** Lisa A. Vrooman, Eric A. Rhon-Calderon, Kashviya V. Suri, Asha K. Dahiya, Yemin Lan, Richard M. Schultz, Marisa S. Bartolomei

**Affiliations:** ^1^ Department of Cell and Developmental Biology, Perelman School of Medicine, Epigenetics Institute, University of Pennsylvania, Philadelphia, PA, United States; ^2^ Division of Reproductive and Developmental Sciences, Oregon National Primate Research Center, Oregon Health and Science University, Beaverton, OR, United States; ^3^ Department of Biology, School of Arts and Sciences, University of Pennsylvania, Philadelphia, PA, United States

**Keywords:** embryo culture, assisted reproductive technologies (ART), preimplantation embryo, placenta, imprinted gene, perinatal outcome

## Abstract

Assisted Reproductive Technologies (ART) employ gamete/embryo handling and culture *in vitro* to produce offspring. ART pregnancies have an increased risk of low birth weight, abnormal placentation, pregnancy complications, and imprinting disorders. Embryo culture induces low birth weight, abnormal placental morphology, and lower levels of DNA methylation in placentas in a mouse model of ART. Whether preimplantation embryos at specific stages of development are more susceptible to these perturbations remains unresolved. Accordingly, we performed embryo culture for several discrete periods of preimplantation development and following embryo transfer, assessed fetal and placental outcomes at term. We observed a reduction in fetal:placental ratio associated with two distinct windows of preimplantation embryo development, one prior to the morula stage and the other from the morula to blastocyst stage, whereas placental morphological abnormalities and reduced imprinting control region methylation were only associated with culture prior to the morula stage. Extended culture to the blastocyst stage also induces additional placental DNA methylation changes compared to embryos transferred at the morula stage, and female concepti exhibited a higher loss of DNA methylation than males. By identifying specific developmental windows of susceptibility, this study provides a framework to optimize further culture conditions to minimize risks associated with ART pregnancies.

## Introduction

Over eight million babies worldwide have been conceived using Assisted Reproductive Technologies (ART). Although the vast majority of babies are born healthy, ART pregnancies are at increased risk for several complications, specifically low birth weight, stillbirth, preterm birth, and abnormal placentation, which includes increased risk for preeclampsia and morbidly adherent placentas (Daniel et al., 1999; Schieve et al., 2007; Wisborg et al., 2010; Haavaldsen et al., 2012; Pandey et al., 2012; Marino et al., 2014; Luke et al., 2017, 2019; Stern et al., 2018; Sacha et al., 2022). ART pregnancies are also at increased risk for rare imprinting disorders that severely affect development (DeBaun et al., 2003; Gicquel et al., 2003; Ludwig et al., 2005; Hiura et al., 2012; Mussa et al., 2017; Cortessis et al., 2018; Johnson et al., 2018; Uk et al., 2018; Hattori et al., 2019a; Henningsen et al., 2020). Studies using a mouse model indicate that some of these adverse effects are driven primarily by embryo culture (Mann et al., 2004; Rivera et al., 2008; Delle Piane et al., 2010; de Waal et al., 2014; Chen et al., 2015; Vrooman et al., 2020). For example, embryo culture induces reduced fetal:placental ratios, placental junctional zone overgrowth, and loss of placental DNA methylation at imprinting control regions (ICRs) in term mouse concepti (Sui et al., 2014; Chen et al., 2015; de Waal et al., 2015; Vrooman et al., 2020).

Embryo culture is intrinsic to ART. For patients undergoing ART procedures, it is not a question of whether to culture embryos, but rather, what culture conditions will be used and when embryos will be transferred to mitigate the aforementioned adverse effects. To date, there is no widely accepted consensus about conditions for embryo culture and transfer for ART, noting that ART procedures have significantly changed during the relatively short history of *in vitro* fertilization (IVF). For example, human embryo transfers were initially performed at the cleavage/morula stage, which allow embryos to return to an *in vivo* environment after 3–5 days of culture. As culture conditions improved to support development to the blastocyst stage, many clinics shifted to extended culture to the blastocyst stage, which requires an additional 2–3 days (Glujovsky et al., 2016). Two advantages of extended culture are that it enables selection of the most developmentally advanced embryos, matching the embryonic stage to when embryos normally arrive in the uterus in spontaneous pregnancies, and genetic testing on biopsied trophectoderm cells rather than earlier stage blastomeres that may affect development of the embryo proper (Neuhausser et al., 2020). Some evidence suggests that blastocyst transfer improves implantation, pregnancy and live birth rates, and reduces the risk for small for gestational age compared to cleavage transfer (Frattarelli et al., 2003; Papanikolaou et al., 2005, 2006; Elgindy et al., 2011; Fernández-Shaw et al., 2015; De Vos et al., 2016; Wang et al., 2017). These findings are controversial, however, because other studies reported that blastocyst transfer pregnancies have a higher risk of placental abnormalities/complications, preterm birth, perinatal mortality, and large for gestational age, as well as skew sex ratios in favor of males compared to cleavage stage transfers (Chang et al., 2009; Kalra et al., 2012; Dar et al., 2013, 2014; Ginström Ernstad et al., 2016; Wang et al., 2017; Hattori et al., 2019b; Spangmose et al., 2020). Such findings raise concern that extended culture may cause unwanted adverse outcomes.

To our knowledge, there are currently no data on the potential for extended culture to induce significant epigenetic changes or adverse long-term health outcomes compared to cleavage stage transfers. Preimplantation development is characterized by critical transitions, any of which could be more susceptible to embryo culture. Following fertilization, both parental genomes undergo a dramatic global erasure of DNA methylation (Messerschmidt et al., 2014). In mouse, major zygotic genome activation occurs during the 2-cell stage (Aoki et al., 1997). Compaction occurs at the 8-cell stage and blastomere polarization is followed by asymmetric cell divisions that generate inner and outer cells of the morula (Mihajlović and Bruce, 2017). The outer cells of the morula will become trophectoderm, which gives rise to the extraembryonic tissues. These events are followed by cavitation and formation of blastocysts with a distinct trophectoderm and inner cell mass (ICM). The ICM remains pluripotent and will give rise to embryonic tissues (Cockburn and Rossant, 2010). Although some *de novo* DNA methylation initiates in the ICM, the majority of DNA methylation happens post-implantation (Auclair et al., 2014). Failure to execute any of these steps in a timely or accurate manner can result in non-viable embryos or embryos at risk for developmental defects. It remains unknown whether specific periods of preimplantation development *in vitro* are more susceptible to embryo culture and what biological processes are adversely affected that could have long-term consequences.

Here we demonstrate that fetal:placental ratio is affected by culture at two distinct windows of preimplantation embryo development, whereas placental morphological abnormalities are only associated with embryo culture prior to the morula stage. Using the Infinium Mouse Methylation BeadChip, we also show that embryo culture-induced loss of placental DNA methylation at ICRs occurs prior to the morula stage, but extended culture to the blastocyst stage induces additional hypomethylation. Although both male and female concepti exposed to embryo culture exhibit reduced global methylation compared to unexposed concepti, female concepti exhibit a higher loss of global DNA methylation than males following culture.

## Materials and Methods

### Animals

Breeding stocks of CF-1 female mice (Envigo, Wilmington, MA, United States), B6SJLF1 (The Jackson Laboratory, Bar Harbor, ME), and CD-1 (Charles River, Indianapolis, IN) male mice were maintained in a pathogen-free facility. All animals were housed in polysulfone cages and had access to drinking water and chow (Laboratory Autoclavable Rodent Diet 5010, LabDiet) ad libitum.

### Generation of Natural and Control Concepti

Sexually mature CF-1 female mice were used between 2 and 3 months-of-age. Naturally conceived concepti were generated by mating naturally cycling CF-1 females to B6SJLF1 males. The day of a vaginal plug was denoted as embryonic day (E) 0.5 and development occurred *in vivo* without embryo transfer. All experimental culture groups and transfer control concepti were generated by superovulating CF-1 females using standard gonadotropin protocols (Behringer et al., 2014). Briefly, females were injected with 5 IU PMSG at 12 p.m., followed by 5 IU hCG 48 h after PMSG injection. Females were paired with one of six available B6SJLF1 stud males. For the superovulated (S) morula and S blastocyst groups, fertilized eggs developed *in vivo* before being flushed from the oviducts with warm HEPES-buffered minimum essential media (MEM) 72 and 96 h after hCG injection, respectively, just prior to embryo transfer.

### Embryo Culture

For all embryo culture groups, embryos were flushed from the oviducts with warm HEPES-buffered MEM and washed in a minimum of four drops of EmbryoMax KSOM medium (1x) containing ½ amino acids (KSOM + AA, EMD Millipore) covered in mineral oil, and cultured to the appropriate stage in a final KSOM + AA droplet under mineral oil at 37°C in an atmosphere of 5% CO_2_, 5% O_2_, 90% N_2_.

For the one-cell to morula culture group (1-cell-morula), eggs were fertilized *in vivo* and the embryos were flushed from the oviducts 24 h after hCG injection. Embryos were then cultured for 2.75 d prior to transfer. The four-cell to morula culture group (4-cell-morula) contained some four- and five-cell embryos but the vast majority of embryos were six-cells at collection. Embryos were flushed from the oviducts 68 h after hCG injection, washed, and cultured for 24 h before transfer. For morula-blastocyst culture group, embryos were flushed from the oviducts 72 h after hCG injection, and cultured to the blastocyst stage for 24 h before transfer. For the one-cell to blastocyst group (1-cell-blastocyst), embryos were flushed from the oviducts 24 h after hCG injection, and cultured to the blastocyst stage for 96 h before transfer. For all culture groups, embryos were left to develop undisturbed from the beginning of culture until embryo transfer.

### Embryo Transfer

Ten embryos of good morphology were washed with a minimum of four drops of warm HEPES-buffered MEM and transferred to E2.5 (for morula transfer groups) or E3.5 (for blastocyst transfer groups) pseudopregnant recipients using non-surgical embryo transfer (NSET) devices (Paratechs).

### Tissue Collection

Concepti were collected at E18.5. Embryonic day was determined by day of vaginal plug (E0.5) for spontaneously pregnant Natural females or the day of embryo transfer (E2.5 for morula transfers or E3.5 for blastocyst transfers). Concepti were collected 17.5 days after the presence of a vaginal plug for spontaneously pregnant Natural females, 16.5 days after embryo transfer for morula transfer groups and 15.5 after embryo transfer for blastocyst transfer groups. Fetal and placental wet weights were recorded. The yolk sac from each embryo was collected and processed for sex genotyping. Placentas were cut in half through the umbilical cord attachment site. One half was snap frozen in liquid nitrogen and stored at −80 °C for DNA and RNA isolation and the other half was fixed in 10% phosphate-buffered formalin. Fixed tissues were then processed through an ethanol dehydration series and xylenes prior to embedding in paraffin wax. Tissue sections (5 µm) were prepared for *in situ* hybridization analyses.

All viable concepti (a minimum of 15 per group) from a minimum of five different dams were analyzed for all experiments, except the DNA methylation chip experiment (see ‘Genome-wide DNA methylation profiling’ section for details). Eleven concepti per group provides the power to detect significant differences in fetal weight, placental weight, and loss of imprinting with an alpha = 0.05.

### Sex Genotyping

Sex was determined by reverse transcriptase PCR using primers for *Kdm5c* and *Kdm5d* using genomic DNA isolated from fetal tissue of each conceptus as previously described (SanMiguel et al., 2018).

### *In situ* Hybridization

Antisense and sense control *in situ* hybridization probes were designed, synthesized, and hybridized as previously described (Bany and Simmons, 2014; Vrooman et al., 2020). The antisense *Tpbpa in situ* hybridization probe was denatured at 65 °C for 5 min and applied to placental sections, while sense *Tpbpa in situ* hybridization probe control was added to a negative control slide (100 μg probe in 75 μl of hybridization buffer per placenta section). Tissue sections were covered with warmed glass coverslips, sealed with rubber cement, and incubated overnight at 65 °C in humid chambers. Slides were briefly washed and anti-DIG-AP Fab fragments (Sigma-Aldrich) (1:1,000) were applied to sections for 1 h at room temperature prior to color development using NBT/BCIP stock solution diluted following manufacturer’s instructions (Roche). Slides were counterstained with Harris hematoxylin and mounted using ImmunoHistoMount (Sigma-Aldrich) and imaged using an EVOS FL Auto Cell Imaging System and software (Life Technologies) at 20× magnification. The junctional zone (*Tpbpa*-positive) and whole placenta area, excluding residual decidua, were measured using FIJI (ImageJ v2.1.0, National Institutes of Health) by at least two individuals blinded to the experimental group. The percentage of junctional zone present was calculated as the total junctional zone area/whole placenta area (excluding decidua) 100×. Parietal trophoblast giant cells were identified by their distinct morphology and location between the junctional zone and decidua and counted by at least individuals blinded to the experimental group. Junctional zone area measurements and pTGC counts for each placenta are an average of the individual scores.

### DNA and RNA Isolation

DNA and RNA were simultaneously isolated from one-quarter of each snap-frozen placenta as previously described (de Waal et al., 2014).

### Bisulfite Pyrosequencing Assay

DNA methylation at *H19/Igf2*, *Kcnq1ot1*, and *Peg3* ICRs was measured using bisulfite-treated DNA as previously described (de Waal et al., 2014) and performed by individuals blinded to experimental group.

### Genome-wide DNA Methylation Profiling

Bisulfite-treated DNA (1,000 ng) was pipetted onto an Illumina Infinium Mouse Methylation-12v1-0 BeadChip (Illumina, CA, United States), and run on an Illumina iScan System (Illumina, CA, United States) using the manufacturer’s standard protocol. The samples were processed at the Center for Applied Genomics Genotyping Core at the Children’s Hospital of Philadelphia. The Illumina Infinium Mouse Methylation-12v1-0 BeadChip includes 284760 CG probes, 1,352 high-frequency SNP probes (rs probes), and 938 non-CpG targeting probes (Ch probes) (287,050 probes total) as defined in “Infinium Mouse Methylation v1.0 A1 GS Manifest File. bpm” (Zhou et al., 2022).

Bisulfite converted placental DNA samples were chosen from the following experimental groups: natural pregnancies (n = 10), 1cell-morula (n = 12), and 1cell-blastocyst (n = 12). One male and one female placenta were selected from each litter. In cases where more than one female or male littermate sample was available, the littermate closest to the litter mean for placental weight was chosen. For male 1cell-morula and 1cell-blastocyst placentas, five samples came from different litters and the sixth male was a littermate chosen based on which male sample was closest to the group mean for all males within the experimental group.

### Genome-wide Methylation Analyses

Raw IDAT files were processed with SeSAMe R package (v1.10.4) and the MM285 array manifest file that comes with the package (vM25). Briefly, SeSAMe sets quality mask for probes, computes detection *p*-value using out-of-band probes empirical distribution and produces methylation β-values, which were used for all downstream analyses. The MM285 array manifest file is the annotation of the Infinium DNA Methylation BeadChip probes. This annotation includes basic probe design information such as mapping location, Infinium-I versus Infinium-II chemistry, target sequence context, tango address on the array, probe sequence, design principle, probe exclusion lists (masks), gene association, overlap with epigenomic features. We used “Infinium Mouse Methylation v1.0 A1 GS Manifest File. bpm” manifest for processing MouseMethylation-12v1-0 data. Nearby genes were assigned to probes based on proximity using the annotatr R package (Cavalcante and Sartor, 2017). All downstream analysis was conducted using the mm10/GRCm10 mouse genome assembly.

Differentially methylated regions (DMRs) were identified using the bumphunter R package included in minfi package (Jaffe et al., 2012). We performed 100 permutations using a maximum pairwise distance of 250 bp and used the 95th percentile of calculated effect size estimates as the threshold (coef = 1, cutoff = 0.1, B = 100, maxGap = 250, pickCutoff Q = 0.95). DMR annotations were obtained using the annotate Peaks function of the R package, ChIPSeeker (Yu et al., 2015) (version 1.22.1 parameter: TxDb = TxDb.Mmusculus.UCSC.mm10. knownGene). Venn diagrams were obtained using the package VennDiagram for R. We overlapped data sets using gene names for hypomethylated DMRs but the package considers multiple DMRs present in one gene as one. Additional annotations and chromosomal distributions were obtained using annotatr package for R with mm10/GRCm10 mouse genome assembly. We used the DMRs from bumphunter analysis as input dataset and obtained as output a list of predicted genomic regions in which you can find these DMRs. The number of identified regions were higher than our number of DMRs because some DMRs overlap with more than one genomic locus. Pathways analyses were performed using PathfindR (Ulgen et al., 2019) and tissue enrichment analysis using the TissueEnrich R package (Jain and Tuteja, 2019). Bedtools Fisher (v2.30.0) was used to perform a Fisher’s exact test of overlap between affected DMRs and promoters and intronic regions to determine an odds ratio (Quinlan and Hall, 2010). We used the proportion of the genome each region comprises and chromosomal size to determine whether the degree of overlap observed is more or less than expected. Bedtools Fisher test was also used to calculate the odds ratio of having DMRs containing repetitive elements for males and females together and separately.

### Statistics

For end points that can be influenced by dam variation and litter size (fetal weight, placental weight, fetal placental ratio, pTGC counts, and junctional zone measurements), we fit a mixed effects model to the data, including the embryo culture group as the fixed effect and dam as the random effect. The model was refit without embryo culture groups, and a likelihood ratio test used to globally assess evidence for differences in means among the culture groups. If the global test yielded a *p*-value less than the type I error rate of 0.05, we then used pairwise contrasts of the model coefficients to identify pairs of groups with different means. The pairwise tests were adjusted for multiplicity using a Holm-Bonferroni correction in order to strictly maintain the family-wise type I error rate at 0.05. Mixed effects models were fitted using package (nlme) in R v 3.6.2 (R foundation for Statistical Computing; www.R-project.org/). DNA methylation of imprinted genes is not subject to influence from the dam or litter size, thus one-way ANOVA and variance ratio tests and were used to determine if significant differences in the mean and variance in DNA methylation from bisulfite pyrosequencing assays differed among groups. One-way ANOVA and variance ratio tests were performed using GraphPad Prism version 9.1.1 and groups were noted as statistically significant of *p* < 0.05.

### Study Approval

All animal work was conducted with the approval of the Institutional Animal Care and Use Committee and the University of Pennsylvania.

## Results

**FIGURE 1:**
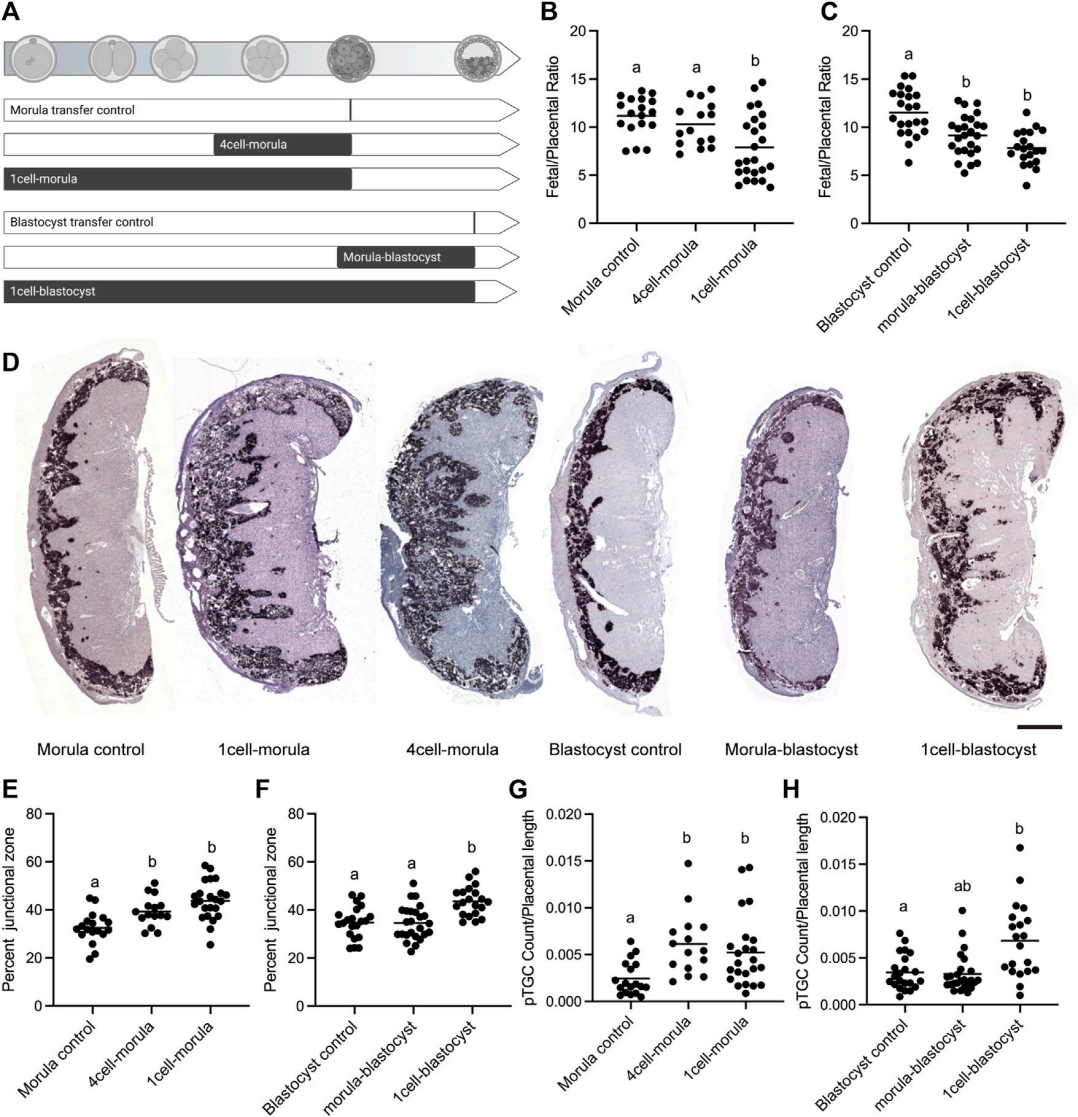
Fetal and placental outcomes in E18.5 mouse concepti after different periods of embryo culture. **(A)** Embryo culture experimental paradigm. White and black bars denote timing of *in vivo* and *in vitro* culture development for each group, respectively. Refer to Methods for details. **(B–C)** Fetal/placental ratio of morula and blastocyst transfer groups. **(D)** Representative images of placental cross-sections for morula and blastocyst transfer groups after Tpbpa *in situ* hybridization, counterstained with hematoxylin. Scale bar = 1 mm. **(E–F)** Junctional zone area as a percentage of the total placental area based on Tpbpa staining for morula and blastocyst transfer groups. **(G–H)** Parietal trophoblast giant cell counts normalized to placental length for morula and blastocyst transfer groups. All concepti were analyzed and each data point represents an individual conceptus. Concepti from a minimum of five different litters were analyzed for each experimental group (n = 15–25 per group). Black line represents the mean of each group. A significant (*p* < 0.05) global likelihood ratio test of differences between groups in the mixed effects model was followed by pairwise tests adjusted using a Holm-Bonferroni correction. Groups with different letters indicate significant differences between groups (adjusted *p* < 0.05); the same letters indicate no difference was detected.

We assessed the morphological and epigenetic impact of discrete windows of embryo culture that coincide with distinct biological processes during preimplantation development (Figure 1A). The 4-cell-morula group (24 h total culture time) targets numerous processes, including compaction, blastomere polarization, asymmetric cell divisions, lineage segregation, and the last phase of global DNA demethylation. The 1-cell-morula group (2.75 d total culture time) covers these processes as well as the entire period of global DNA demethylation and zygotic genome activation. This window is clinically relevant to cleavage-stage transfers in human IVF cycles. The morula-blastocyst group (24 h total culture time) targets *de novo* DNA methylation, cavitation, and further differentiation of the trophectoderm. The maximal culture window (1-cell-blastocyst group) captures all preimplantation development prior to transfer and is relevant to blastocyst transfer in human IVF cycles.

To match the *in vivo* synchrony between stage of embryo development and uterine receptivity, control morula, 4-cell-morula, and 1-cell-morula embryos were transferred to pseudopregnant females 2.5 days after vaginal plug detection, whereas control blastocyst, morula-blastocyst, and 1-cell-blastocyst embryos were transferred to pseudopregnant females 3.5 days after vaginal plug detection. Concepti were assessed at term (E18.5) for fetal:placental ratio, placental morphology, and DNA methylation levels, end points with robust phenotypes previously observed in mouse concepti exposed to embryo culture (Chen et al., 2015; de Waal et al., 2015; Vrooman et al., 2020).

### Fetal Weight is Impacted by Culture at Two Independent Windows in Preimplantation Embryo Development

Embryo culture, even under optimized conditions (5% oxygen and amino acid supplemented culture medium), can reduce fetal weight and induce placental overgrowth in term mouse concepti (Chen et al., 2015; de Waal et al., 2015; Vrooman et al., 2020). To determine if these growth abnormalities were associated with culture during a specific time of preimplantation development, term mouse concepti that were cultured for different periods of preimplantation development were assessed. For morula transfer groups, fetal weight at term was significantly reduced only in the 1-cell-morula group compared to controls, whereas placental weight was significantly increased in both the 1-cell-morula and 4-cell-morula groups compared to controls (Table 1). For blastocyst transfer groups, fetal weight was significantly reduced in both the 1-cell-blastocyst and morula-blastocyst groups, whereas placental weight was significantly increased in only the 1-cell-blastocyst group compared to blastocyst controls (Table 1).

**TABLE 1:** Fetal weight, placental weight, and fetal/placental ratio of mouse concepti exposed to embryo culture during preimplantation development.

	Fetal Weight (g)	Placental Weight (g)	Fetal/Placental ratio
Natural^a^	1.361 ± 0.015	0.097 ± 0.002	14.51 ± 0.39
Morula control	1.503 ± 0.029 a	0.139 ± 0.006 a	11.18 ± 0.48a
4 cell-morula	1.645 ± 0.063 a	0.164 ± 0.007 b	10.29 ± 0.59 a
1 cell-morula	1.300 ± 0.055 b	0.183 ± 0.011 b	7.90 ± 0.69 b
Blastocyst control	1.417 ± 0.029 a	0.127 ± 0.005 a	11.53 ± 0.51 a
Morula-blastocyst	1.167 ± 0.029 b	0.133 ± 0.006 a	9.10 ± 0.42 b
1 cell-blastocyst	1.216 ± 0.041 b	0.159 ± 0.005 b	7.82 ± 0.40 b

a Natural group is shown for visual comparison but was not statistically tested.

A significant (*p* < 0.05) global likelihood ratio test of differences between groups in the mixed effects model was followed by pairwise tests adjusted using a Holm-Bonferroni correction. Groups with different letters indicate significant differences between groups (adjusted *p* < 0.05); the same letters indicate no difference was detected.

Fetal/placental ratio can be used as a rough measure of placental efficiency, with lower fetal/placental ratios associated with a defect in placental function (Hayward et al., 2016). Embryo culture from the 1-cell-blastocyst stage reduces fetal/placental ratio at term in mice compared to groups that were not exposed to embryo culture (Chen et al., 2015; de Waal et al., 2015; Vrooman et al., 2020). To determine if reduced term fetal/placenta ratios are linked to a specific time of embryo culture, we assessed fetal/placenta ratios among transfer groups. Among groups transferred at the morula stage, only the 1-cell-morula culture group had significantly reduced fetal/placental ratios compared to the morula transfer control (Figure 1B). This difference is contributed by both significantly smaller fetuses and larger placentas than controls. Among groups transferred at the blastocyst stage, both the 1-cell-blastocyst group and the morula-blastocyst group had significantly reduced fetal/placental ratios compared to blastocyst transfer controls (Figure 1C). Although the 1-cell-blastocyst group has both significantly smaller fetuses and larger placentas than controls, the difference in fetal/placental ratio in the morula-blastocyst group is only contributed by significantly smaller fetuses, and no significant change in placental weight compared to controls.

These findings suggest both the 1-cell-morula and morula-blastocyst culture windows independently impact fetal/placental ratios but through different mechanisms. Both windows reduced fetal weight, but only the 1-cell-morula culture also affected the placenta. Moreover, any exposure to culture appears to disrupt the uniform and predictable relationship of fetal weight and placental weight in each individual conceptus (Supplementary Figure S1). As we have observed before (Vrooman et al., 2020), both males and females are equally affected by embryo culture (Supplementary Figure S2A,B), although we may be underpowered to detect subtle sex differences. These effects were not associated with any significant differences in litter size, implantation rate, live concepti rate, or sex ratio (Supplementary Table S1).

### Placental Overgrowth is Induced by Embryo Culture Prior to the Morula Stage

The junctional zone—the placental compartment that produces hormones that influence and maintain pregnancy—is disproportionately enlarged in term IVF placentas (Sui et al., 2014; Chen et al., 2015; de Waal et al., 2015; Vrooman et al., 2020) due to an overrepresentation of glycogen trophoblasts and parietal trophoblast giant cells (pTGCs) induced by embryo culture procedures (Vrooman et al., 2020). To assess if this junctional zone overgrowth is attributed to a discrete window of preimplantation development, we visualized the junctional zone area using *in situ* hybridization of placental sections with an antisense probe for the junctional zone-specific marker *Tpbpa* in the various culture groups (Figure 1D) (Simmons, 2014). For morula transfer groups, embryos cultured from both the 1-cell-morula stage and 4-cell-morula stage showed significant junctional zone overgrowth compared to controls, but were not significantly different from each other (Figures 1D,E). For blastocyst transfer groups, only the 1-cell-blastocyst culture group had significant junctional zone overgrowth compared to controls (Figures 1D,F). Embryos cultured from both the 1-cell-morula stage and 4-cell-morula stage also showed a significant increase in the number of pTGCs compared to controls, but were not significantly different from each other (Figure 1G). For blastocyst transfer groups, only the 1-cell-blastocyst culture group showed a significant increase in the number of pTGCs compared to controls (Figure 1H). Females had more junctional zone overgrowth in the 4-cell-morula group as well as an increase in pTGCs in the 1-cell-blastocyst group compared to males in the same experimental group (Supplementary Figure S2C,D).

In summary, a 24-h culture that encompasses development from the 4-cell to morula stage leads to placenta overgrowth and the disproportion of junctional zone and pTGCs in mouse placenta. This finding was unexpected because we suspected that placental phenotypes would be associated with morula to blastocyst culture, as this timing coincides with trophectoderm differentiation. Thus, the difference in placental morphology with embryo culture is likely a result of disrupting early trophectoderm differentiation-related gene expression or asymmetric division regulation prior to the morula stage.

### Significant Loss of DNA Methylation at Imprinting Control Regions is Associated With Embryo Culture Prior to the Morula Stage

**FIGURE 2:**
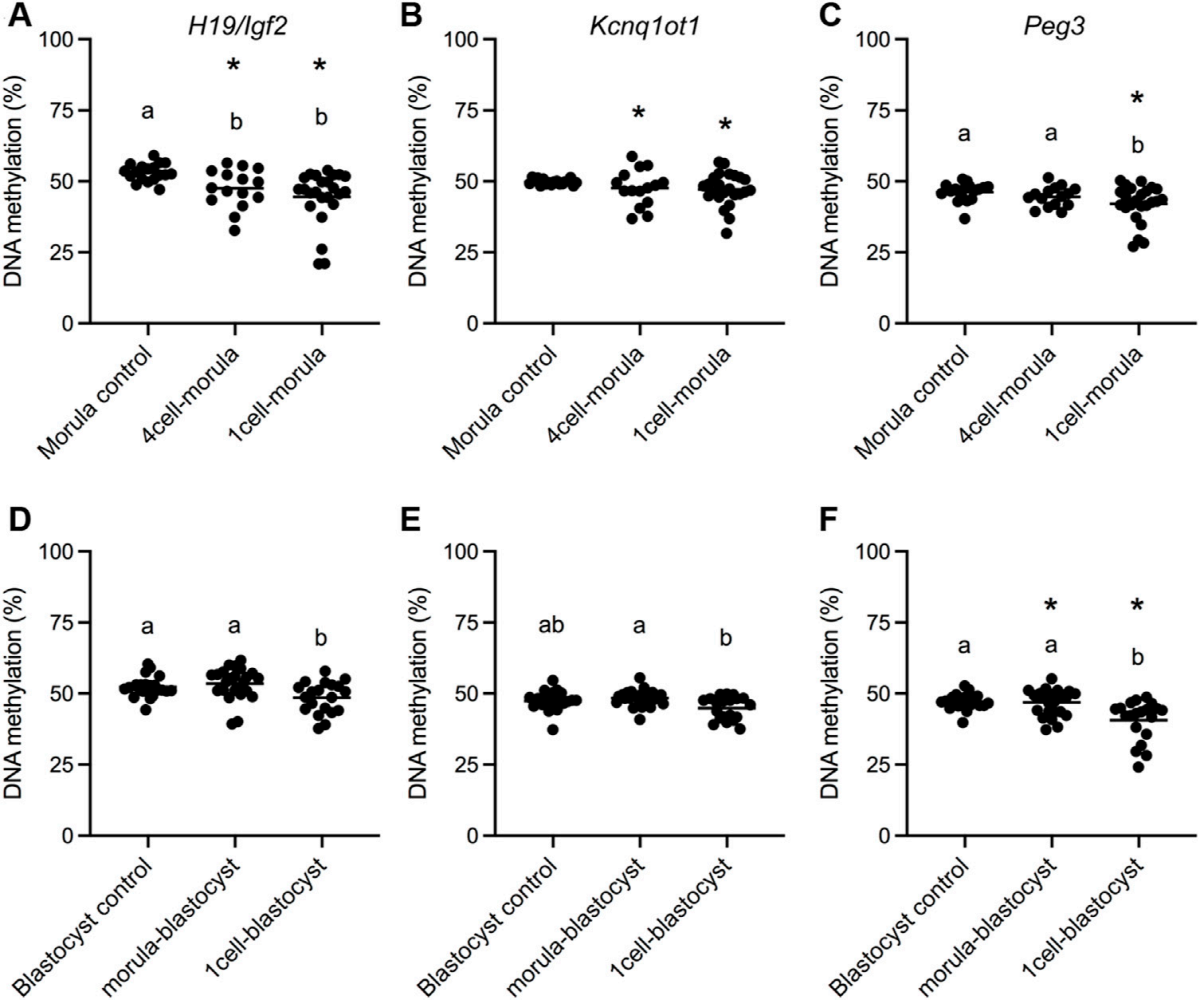
DNA methylation status in E18.5 placentas after different periods of embryo culture during preimplantation development. Bisulfite pyrosequencing was conducted on E18.5 placentas for **(A)** *H19/Igf2* ICR, **(B)** *Kcnq1ot1* ICR and **(C)** *Peg3* ICR morula transfer groups and **(D)** *H19/Igf2* ICR, **(E)** *Kcnq1ot1* ICR and **(F)** *Peg3* ICR blastocyst transfer groups. All concepti were analyzed and each data point represents an individual placenta from a minimum of five different litters (n = 15–25 per group). Black line represents the mean of each group. Statistical significance of imprinted gene assays was determined by both one-way ANOVA for differences in the mean and F-ratio test for differences in variability. Groups with different letters indicate significant differences between means (*p* < 0.05); the same letters indicate no difference in means was detected. Groups with asterisks have significantly different variability from groups with no asterisks (*p* < 0.05).

Imprinted genes are expressed in a parent-of-origin specific manner and regulated by differential DNA methylation at ICRs, which is inherited from gametes and maintained in preimplantation embryos (Barlow et al., 2014). Both mouse and human embryos conceived by IVF display loss of DNA methylation at ICRs (10–25). We previously demonstrated that the paternally methylated ICR, *H19/Igf2*, and two maternally methylated ICRs, *Kcnq1ot1* and *Peg3*, are consistently hypomethylated in mouse placental tissues and this effect is caused by embryo culture (Mann et al., 2004; Rivera et al., 2008; de Waal et al., 2014, 2015; Chen et al., 2015; Vrooman et al., 2020). Because differences in mean ICR methylation can be driven by a subset of affected concepti, we assessed the various culture groups for ICR methylation differences for both mean and variance (de Waal et al., 2014, 2015). As we and others have previously observed (Mann et al., 2004; Rivera et al., 2008; de Waal et al., 2014, 2015; Chen et al., 2015; Vrooman et al., 2020), all three ICRs had reduced mean DNA methylation and/or increased variance in the 1-cell-blastocyst culture group (Figures 2D–F). For the 1-cell-morula culture group, both reduced mean DNA methylation and increased variance compared to controls were observed for all ICRs (Figures 2A–C). In contrast, the 4-cell-morula group exhibited reduced mean DNA methylation and increased variance for *H19/Igf2*, increased variance only for *Kcnq1ot1*, and no differences in *Peg3* DNA methylation. For the morula-blastocyst culture group, an increase in variance was observed for *Peg3*, whereas no other significant differences were observed at other loci (Figures 2D–F). There were no significant differences between males and females within each group for *H19* and *Kcnq1ot1* ICRs (Supplementary Figure S2E–H). For *Peg3*, however, males in the 4-cell-morula group were hypomethylated compared to females, and females were hypomethylated compared to males in the 1-cell-blastocyst group (Supplementary Figure S2I,J). Taken together, these findings suggest that embryos are only susceptible to loss of ICR DNA methylation in the placenta when cultured prior to the morula stage.

### Loss of Global Placental DNA Methylation is Associated With Time in Culture

**FIGURE 3:**
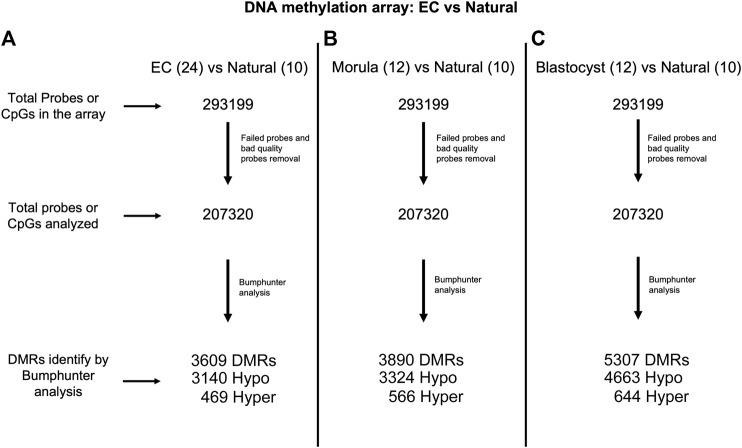
Flow diagram showing probe analysis of the Infinium Mouse Methylation BeadChip. **(A)** Embryo culture (EC) includes both 1-cell-morula and 1-cell-blastocyst groups (n = 24) versus Natural (n = 10). **(B)** 1-cell-morula (n = 12) versus Natural (n = 10). **(C)** 1-cell-blastocyst group (n = 12) versus Natural (n = 10).

Because imprinted genes have one hypermethylated allele that is specifically protected from demethylation during preimplantation development, we were interested in determining if other sequences in the placental epigenome were affected by embryo culture. Genome-wide DNA methylation was measured using the newly developed Infinium Mouse Methylation BeadChip array, which interrogates over 285,000 methylation sites in the mouse genome, providing greater coverage than previously employed. We assayed ten placentas (five males and five females) from naturally conceived pregnancies, 12 placentas (six males and six females) from the 1-cell-morula group and 12 placentas (six males and six females) from the 1-cell-blastocyst group. After obtaining the raw data and QC files for each individual, we first removed the randomly designed and failed probes, resulting in 200,000 probes for our analyses (Figure 3). Because of the limited sample size of the groups and our interest in assessing sex-specific effects, we did not have the statistical power to detect significant changes in methylation probe by probe. Instead, we took advantage of the available R package, Bumphunter, part of the Minfi R package, which allows identification of differentially methylated regions (DMRs) by bumps or peaks of DNA methylation affected by the length of culture, using a small number of concepti per group (Jaffe et al., 2012).

Previous work by Salilew-Wondim and collaborators showed that the length of embryo culture could impact genome-wide DNA methylation of blastocysts in a bovine model (Salilew-Wondim D, et al., 2018, 2015). They observed major changes in hypermethylated DMRs in embryos cultured to the morula and blastocyst stage. These contrasting results could be due to a number of differences between studies, including the species (mouse vs bovine), methods (*in vitro* culture of embryos using optimized KSOM + AA medium vs bovine studies that require *in vitro* maturation of oocytes prior to fertilization using different culture medium), tissues analyzed (blastocysts, which are relatively hypomethylated vs term placenta, which has more global methylation than blastocysts), as well as the analysis (DMRs) as a “bump” or peak (see bumphunter analysis in methods vs considering single probes as DMRs).

**FIGURE 4:**
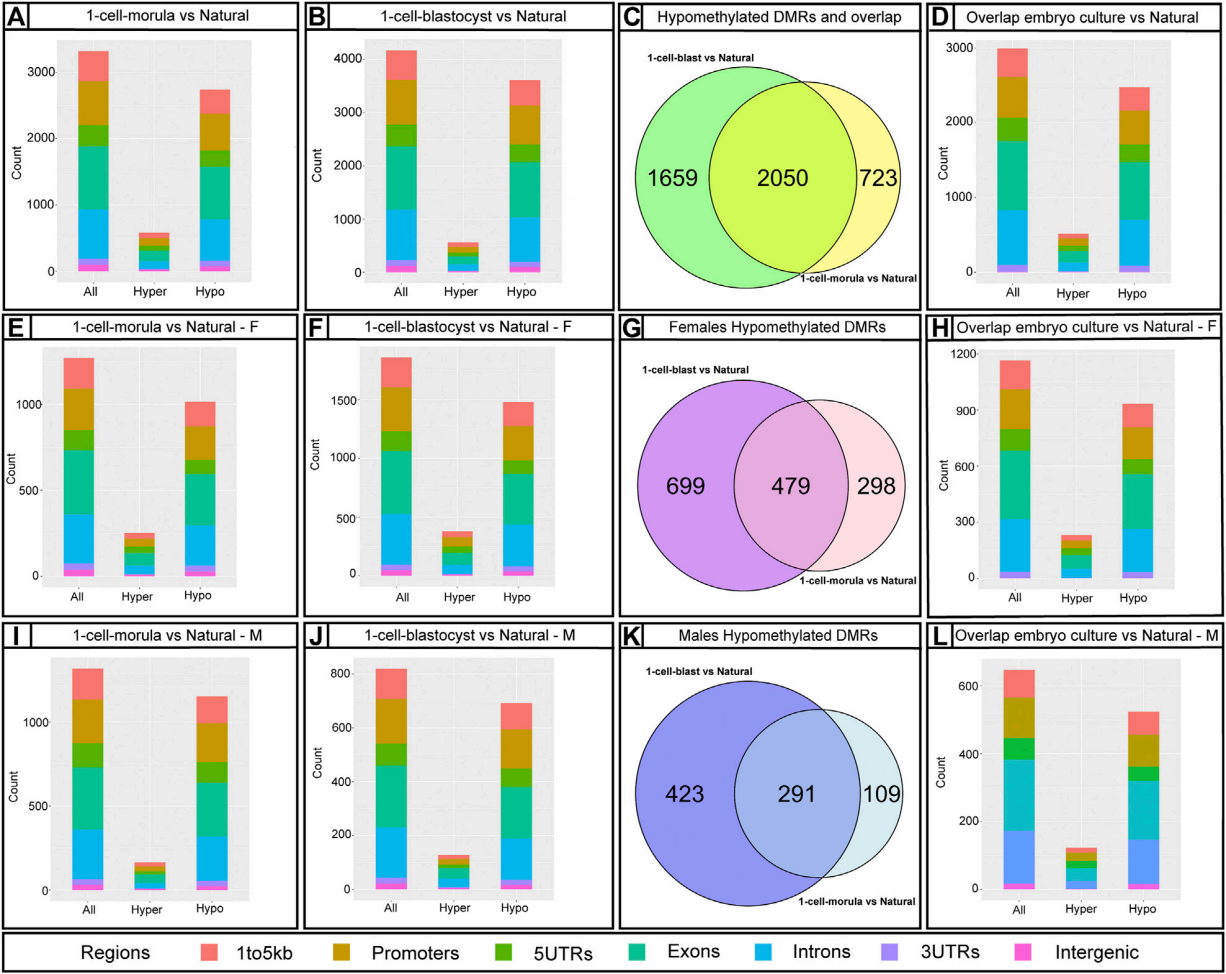
Infinium Mouse Methylation BeadChip results using Bumphunter showing the number of annotated DMRs affected by embryo culture and their genomic location. Bar graphs showing annotated DMRs by predicted genomic regions using annotatr R package: **(A,E,I)** 1-cell-morula versus Natural for all, for females **(F)** and males (M). **(B,F,J)** 1-cell-blastocyst versus Natural for all, for females **(F)** and males (M) and **(D,H,L)** overlap between 1-cell-morula versus Natural and 1-cell-blastocyst versus Natural for all, for females **(F)** and males (M) placentas. Bar graphs were done using mm10/GRCm10 mouse genome assembly. The number of identified regions were higher than our number of DMRs because some DMRs overlap with more than one genomic locus. Venn diagrams for Hypomethylated DMRs using package VennDiagram for R (this package considered multiple DMRs in a gene as one): **(C,G,K)** 1-cell-morula versus Natural and 1-cell-blastocyst versus Natural to find overlap between the two analyses for all, for females and males. For all analyses, 1-cell-morula (n = 12, six females and six males), 1-cell-blastocyst (n = 12, six females and six males) and Natural (n = 10, five females and five males).

Bumphunter analysis revealed 3,890 DMRs in 1-cell-morula placentas and 5307 DMRs in 1-cell-blastocyst placentas when compared to placentas following natural conception. These DMRs were mapped to genes, resulting in 3,172 and 4,140 affected genes corresponding to the DMRs in 1-cell-morula and 1-cell-blastocyst placentas, respectively. The majority of the DMRs are hypomethylated-- 3,324 and 4,663 in the 1-cell-morula and 1-cell-blastocyst groups, respectively, whereas the rest of the DMRs are hypermethylated, 566 in 1-cell-morula and 644 in 1-cell-blastocyst groups. These results suggest that the DNA methylation that is lost as a consequence of preimplantation development to the morula stage was never recovered and additional methylation loss occurred in embryos that were cultured for an extra day to the blastocyst stage. In comparison to reduced methylation, hypermethylation accounts for only 10–15% of the DMRs and was not altered with extended culture, suggesting that embryo culture had minimal impact on hypermethylation relative to hypomethylation and that mechanisms exerting those changes were confined to development prior to the morula stage. In accordance with the targeted pyrosequencing data, we detected several DMRs, mostly hypomethylated, associated with imprinted genes (Supplementary Table S2). Using the R package, Annotatr (see *Methods and Materials*), we determined that the majority of DMRs are located in promoters and intronic regions and the rest are distributed across other genomic regions (Figure 4). The odds ratio—a means to determine if DMRs are more likely located in promoters than by chance (randomly picking the same number of regions anywhere in the genome)—is similar in both is similar in both 1-cell-morula versus natural and 1-cell-blastocyst versus natural when we analyzed all the data together (Supplementary Table S3). When the data were analyzed by sex, we found that the odds ratio is higher in males compared to females, and the highest ratio is observed in the 1-cell-blastocyst versus natural comparison (Supplementary Table S3). For intronic regions, the odds ratio is similar in both 1-cell-morula versus natural and 1-cell- blastocyst versus natural when we analyzed all the data together and there is not a higher chance to find DMRs in intronic regions and no sex specific effect was observed (Supplementary Table S3).

### DNA Methylation of Repetitive Elements is Altered and Associated With Time in Culture

**FIGURE 5:**
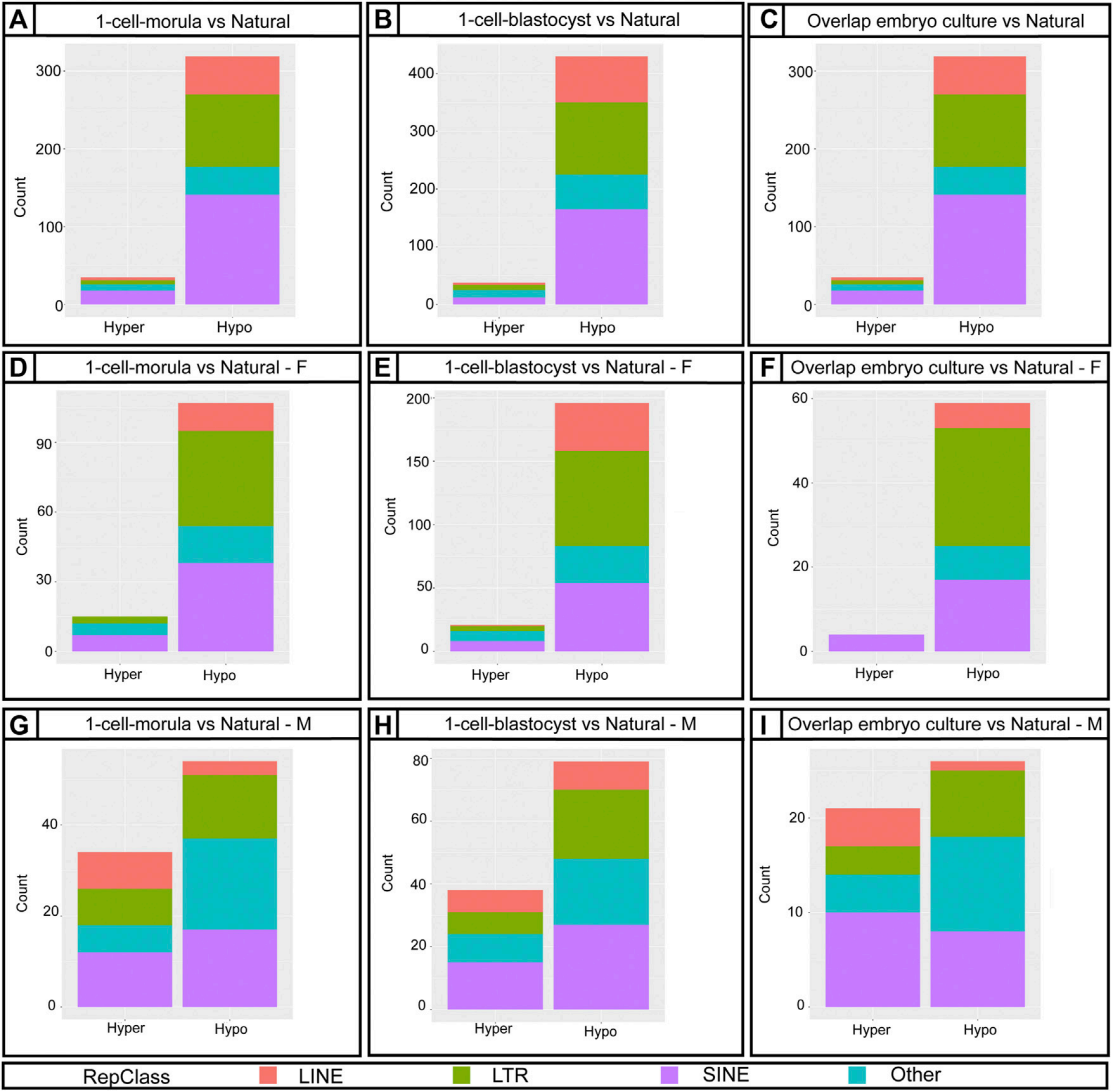
Distribution of annotated DMRs from the Bumphunter results using Repetitive Elements classes. **(A,D,G)** Bar graph showing 1-cell-morula versus Natural for all, for females **(F)** and males (M). **(B,E,H)** Bar graph showing 1-cell-blastocyst versus Natural for all, for females **(F)** and males (M). **(C,F,I)** Bar graph of the overlap between 1-cell-morula versus Natural and 1-cell-blastocyst versus Natural for all, for females **(F)** and males (M). For all analyses, 1-cell-morula (n = 12, six females and six males), 1-cell-blastocyst (n = 12, six females and six males) and Natural (n = 10, five females and five males). For Bumphunter analyses, cutoff value = 0.1. For more details on the parameters used for Bumphunter, refer to *Materials and Methods*. Repetitive classes were found using RepeatMasker tools.

Repetitive elements in the genome are major regulators of gene expression and chromatin organization (Lu et al., 2020). Similar to imprinted genes, repetitive elements are also regulated by DNA methylation (Jones, 2012). To assess DNA methylation changes in repetitive elements, we used the RepeatMasker database downloaded from the UCSC genome browser to identify probes associated with repetitive elements on the Infinium Mouse Methylation BeadChip array. We found there is a higher ratio of DMRs with repetitive elements at promoter regions in 1-cell-blastocyst group than would be expected (Supplementary Table S3). When we analyzed by sex, males had higher odds to have DMRs with repetitive elements at promoters than females. Given that long terminal repeats (LTRs), short interspersed nuclear elements (SINEs) and long interspersed nuclear elements (LINEs) are the most characterized and well-conserved repetitive elements, it was not surprising that the array is enriched for probes targeting these elements (Supplementary Figure S3). The DMRs affected by embryo culture were highly enriched for repetitive elements, mostly LTRs, SINEs, and LINEs, with some other families at a lower percentage (Figure 5). Thus, embryo culture to the morula stage affected repetitive element methylation levels in the placenta compared to embryos that were not cultured. Furthermore, the number of affected elements was higher with culture to the blastocyst stage compared to culture to the morula stage, suggesting that continued culture further exacerbates hypomethylation in this part of the genome (Supplementary Table S14, 15, Figures 5A,B).

### Female Concepti Have Greater Loss of Genome Wide Placental DNA Methylation Than Males

There is evidence that male preimplantation embryos have different developmental trajectories than female embryos (Avery et al., 1991; Valdivia et al., 1993; Bernardi and Delouis, 1996; Bronet et al., 2015; Roos Kulmann et al., 2021) and that placental gene expression and DNA methylation are sexually dimorphic (Martin et al., 2017; Mayne et al., 2017; Gong et al., 2018; Gonzalez et al., 2018; Sun et al., 2020; Braun et al., 2021). Although both male and female concepti are affected by embryo culture with respect to perinatal outcomes, junctional zone overgrowth, pTGC number, and ICR methylation levels compared to controls, we wanted to control for existing sex-specific differences in placental DNA methylation. To this end, we conducted separate placental DNA methylation analyses for males and females. We found that females had more affected DMRs than males—880 hypomethylated and 209 hypermethylated DMRs were found in female placentas from the 1-cell-morula group, whereas males had 448 hypomethylated and 145 hypermethylated DMRs compared to natural conceived placentas of the same sex (Supplementary Table S9, 10). In the 1-cell-blastocyst groups, 1,331 DMRs were hypomethylated and 301 were hypermethylated in the female placentas, compared to natural conceived placentas of the same sex whereas males had 795 hypomethylated and 198 hypermethylated DMRs compared to natural conceived placentas of the same sex (Supplementary Table S12, 13). To determine overlap of affected loci, we compared hypomethylated DMRs between the two sexes. Of those hypomethylated DMRs in both culture groups, only 176 DMRs were shared between males and females in the 1-cell-morula and only 387 DMRs were shared in 1-cell-blastocyst group (Supplementary Figure S4). Repetitive elements were also more affected in females compared to males (Figures 5E–J), again mainly LTRs, SINEs and LINEs. Our data analysis does not support the conclusion that these changes are solely caused by the additional X chromosome and lack of Y chromosome in females, as the affected loci are widely distributed across all chromosomes (data not shown). Autosomal sex-specific DMRs have been identified in humans (Martin et al., 2017; Gong et al., 2018). Human sex-specific DMRs also show that overall males have higher methylation in placenta than females (Gong et al., 2018; Bozack et al., 2021). Thus, although it is unknown why female placentas appear more affected than males with respect to methylation changes, it is possible that inherent sex-specific differences in methylation levels play a role in the sex-specific response to embryo culture. To determine if sex-specific differences in DMRs are due to differences in the percentage of junctional zone, which is hypomethylated compared to the rest of the placenta (Decato et al., 2017), we compared the percent of junctional zone among embryo cultured samples used for genome-wide methylation analysis. We find that placental samples used for the genome-wide methylation array have comparable percentage of junctional zone, suggesting another reason for the sex-specific difference in DMRs (Supplementary Figure S5).

### Placenta-Specific Genes and Pathways Affected by Embryo Culture

**FIGURE 6:**
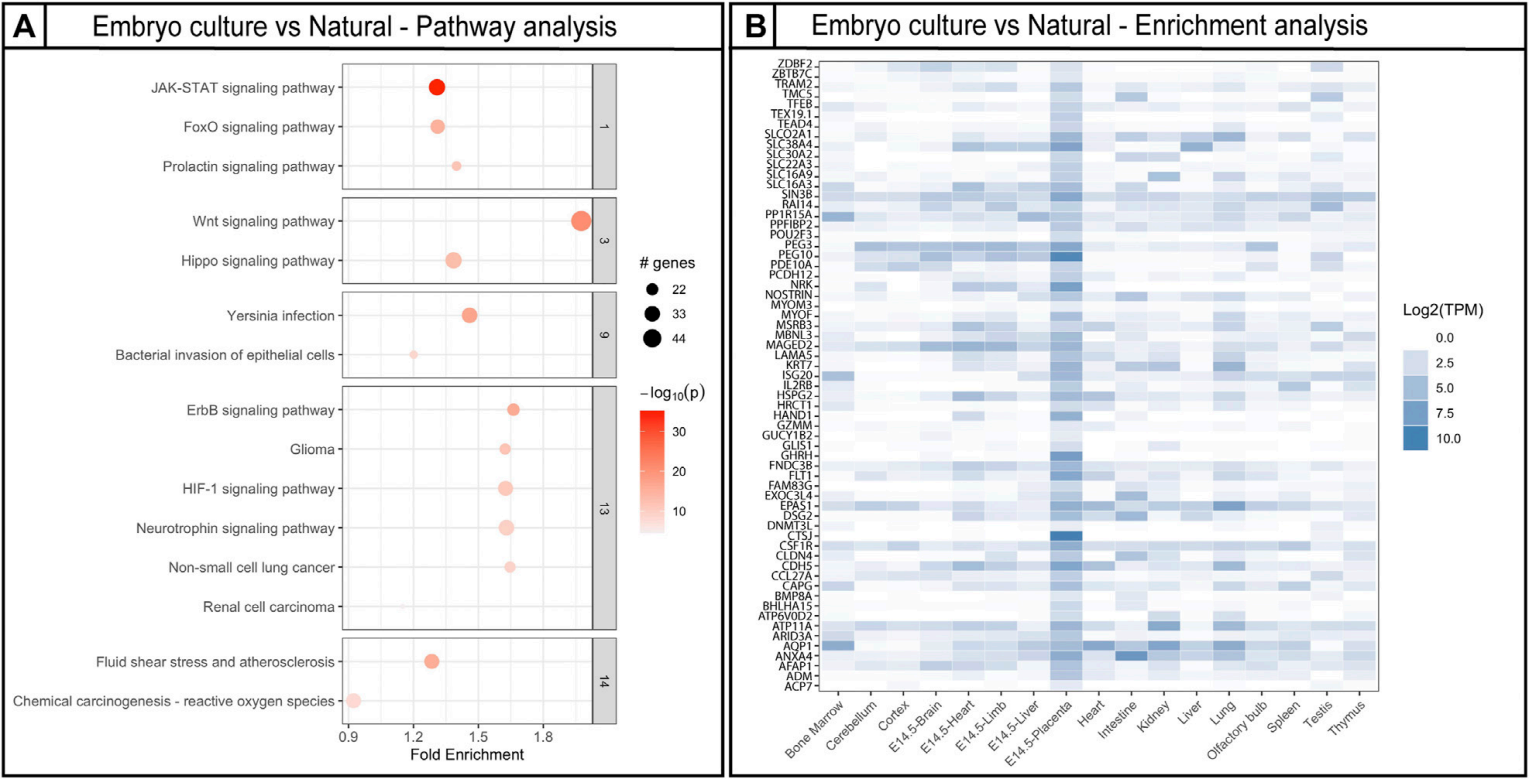
Gene ontology pathway analysis and placenta-specific gene analysis using DMRs affected by embryo culture. **(A)** Top gene ontology pathways affected by embryo culture using genes obtained by Bumphunter. Fold enrichment indicates the ratio of the differentially methylated gene number to the total gene number in a certain pathway. The size of each circle represents the number of genes contained in a particular cluster; a larger circle indicates a larger number of genes. The color indicates the *p*-value (dark orange = high, light orange = low). **(B)** Placental enriched specific genes affected by embryo culture; darker color blue indicates higher number of transcripts in a specific tissue. For Bumphunter analyses, cutoff value = 0.1. For more details on the parameters used for Bumphunter, refer to *Materials and Methods*.

After analyzing placentas from the two different culture window groups independently in comparison to placentas from naturally conceived controls, we identified DMRs shared between the two culture windows. Since loss of methylation has been associated previously with ART, we focus our analysis in comparing the hypomethylated DMRs. Approximately 2050 hypomethylated DMRs were present in both morula and blastocyst groups (Figure 5C). The DMRs were located mainly at intronic and promoter regions (Figure 5D). Given that changes in methylation could potentially impact gene expression and normal placental function, we employed the R Package, Pathfinder, to identify robustly affected placental pathways using the DMRs common to both morula and blastocyst culture. Multiple pathways involved in placental development and function were identified, including the JAK-STAT, WNT, and HIF-1 signaling pathways (Figure 6A). When DMRs were analyzed by intronic or promoter regions, the same affected pathways were identified (Supplementary Figure S7). Further, using the R Package, TissuEnrich, we compared our dataset with the ENCODE database for mouse placenta to obtain a narrowed list of 102 genes highly expressed and specifically enriched in placenta (Figure 6B, Supplementary Table S4, Dataset S2). This list includes imprinted genes (*Peg3, Peg10, Slc38a4*), genes involved in the VEGF pathway (*Flt1*), and cell or tissue-specific markers in the placenta (e.g., *Cdh5*, *Nrk, Pcdh12, Slc16a3*)*.* Similar to the majority of affected DMRs, these placental enriched genes had mainly hypomethylated DMRs (Supplementary Table S4).

## Discussion

Embryo culture is an intrinsic component of ART, which is associated with a number of placental-related pregnancy complications and offspring outcomes. We find that embryo culture conditions disrupt biological processes in preimplantation embryos that specifically impact placentation and that both morphological changes and loss of DNA methylation are associated with embryo exposure to culture prior to the morula stage.

### The Majority of Placental DNA Methylation Loss During Embryo Culture Coincides With the Normal Window of DNA Demethylation During Epigenetic Reprogramming

Previous studies in human and mouse term placenta from ART groups have shown changes in global DNA methylation by luminometric methylation assay (LUMA) (de Waal et al., 2015; Ghosh et al., 2017; Vrooman et al., 2020). Because LUMA measures a specific, globally distributed sequence, this approach does not identify the diversity of gene sequences or give site-specific information. We circumvented this limitation by using the Ilumina Mouse methylation BeadChip that offers single nucleotide resolution and balanced coverage across different genomic elements with high reproducibility.

We confirm changes in genome-wide DNA methylation, mostly hypomethylation due to embryo culture. Because extended blastocyst culture does not result in any changes in the number of hypermethylated DMRs, changes in gene expression due to hypermethylation are likely not of immediate concern. Culture from the 1-cell to morula stage led to significant loss and increased variability of placental DNA methylation at select ICRs that are comparable to levels observed with culture to the blastocyst stage. Similarly, placentas from the 1-cell-morula group had 46 DMRs or bumps in ICRs obtained by Bumphunter, whereas the blastocyst group had 58 DMRs. Of those affected ICRs, 43 were shared between the two groups (Supplementary Table S3). Thus, the majority of DNA methylation loss at ICRs with culture occurs prior to the morula stage, which coincides with the timing of global DNA methylation erasure. Because DNA methylation at ICRs is typically maintained at this time when most of the genome is normally demethylated, we propose that culture impacts the fidelity of the DNA methylation maintenance machinery, either by loss of factors that specifically recognize ICRs or loss of activity of the DNA methylation complex.

Although we did not detect changes in placental DNA methylation at select ICRs between 1-cell-morula and 1-cell-blastocyst with pyrosequencing, we did detect further global placental DNA hypomethylation with extended culture using the methylation array. With respect to imprinted loci, which should be protected from demethylation, differences in methylation loss among the different culture groups suggest individual ICRs have different predispositions to loss of methylation or different rates of methylation loss. With respect to non-imprinted loci, it is unclear what causes the additional hypomethylation, especially that occurring beyond the expected wave of demethylation, e.g., if it is all inherited from DNA methylation loss occurring during culture, an inability of the placenta to have proper *de novo* methylation in response to culture, or a combination of both mechanisms. It is known that the junctional zone is relatively hypomethylated compare to the labyrinth in mouse (Decato et al., 2017). Therefore, increases in junctional zone representation would result in hypomethylated DMR results. It is possible that some hypomethylation differences between Natural and embryo culture groups are indeed due to this difference in junctional zone representation. However, we believe that DMRs between 1cell-morula vs 1cell-blastocyst groups are unlikely to be attributed to cell composition differences alone, as placentas from 1cell-morula are morphologically indistinguishable from those cultured from 1cell-blastocyst. Further, given that ICR methylation should be consistent regardless of cell type, we have confidence in our conclusion that DMR methylation is not due only to cell composition differences as we robustly observed hypomethylation of ICRs with culture (Supplementary Table S2).

Although we cannot infer the function of the individual DMRs from the methylation array, genome-wide methylation differences, which are mostly due to hypomethylation, are detected in several genes and common pathways associated with placental development and function. For example, Wnt signaling is important for placental formation and vascularization (Sonderegger et al., 2010). The JAK-STAT pathway, which is also affected by embryo culture, plays an important role in cell proliferation, cell survival, and angiogenesis. SOCS3-deficient mice, which have increased JAK/STAT signaling, display a significant reduction of spongiotrophoblasts, increased numbers of pTGCs, and poor labyrinth development (Roberts et al., 2001). Although we did not observe a reduction of spongiotrophoblasts, we did observe an increase in pTGCs in concepti cultured prior to the morula stage. Thus, aberrant levels of these proteins could result in the observed defects in vascularization and disproportionate growth of a subset of placental cells.

### Embryo Culture From One-Cell to Morula Development Induces a Loss of Placental DNA Methylation at Repetitive Elements

Repetitive elements are highly conserved and distributed sequences that constitute more than 30% of the mammalian genome. Although multiple classes, families, and subfamilies have been identified, the main function of repetitive elements is still unknown. These elements are mostly inactive due to their hypermethylated state (74), but they may be activated by changes in DNA methylation and serve as alternative promoters for tissue-specific transcripts that potentially increase transcription from the native promoter with a severe effect on transcription without impacting its function (49, 70). There are no existing studies comparing the effects of ART on repetitive elements by class in placenta.

We find that embryo culture, regardless of the duration, reduces methylation of these elements. In human placentas DNA hypomethylation activates repetitive elements that are normally silenced (Macaulay et al., 2014), and differences of DNA methylation at repetitive elements are associated with birth outcomes, including changes in fetal weight (Wilhelm-Benartzi et al., 2012). Hypomethylation of repetitive elements has also been shown to correlate with higher chromosomal instability like what is observed in cancer (Non et al., 2012). Therefore, it is possible that loss of methylation of these elements could contribute methylation of these elements could contribute to some of the morphological phenotypes we observe, such as changes in fetal weight and overproduction of proliferative and invasive trophoblast cell types.

Because DNA methylation of these elements is regulated differently from ICRs (Emera and Wagner, 2012), we suspect that early exposure to non-ideal culture conditions negatively affects the ability of the embryo to regain and/or maintain methylation of these elements. We find that SINEs, LINEs, and LTRs are the most affected classes and are widely distributed across genomic regions (Supplementary Figure S6). These classes are likely the most affected because the array is enriched for probes that target these elements (Supplementary Figure S2) but also because they are the most abundant elements in the genome (Cohen et al., 2011; Lu et al., 2020) and are closely involved in placental function and development. For example, LINE1 and ERVs are key DMRs for placental function (Vlahos et al., 2019). Ghosh et al. analyzed LINE1 methylation in human term placentas using pyrosequencing and found LINE1 to be hypomethylated in the ART group (Ghosh et al., 2017), which is consistent with the hypomethylation of these elements in our mouse model.

Sex differences in the placental methylome have been observed before, but these differences are mainly attributed to the extra X chromosome in females or the presence of Y chromosome in males (Mayne et al., 2017). Although LINE methylation could be impacted by sex, mostly in females due to changes in dietary and hormonal patterns and to a lesser degree in males due to inflammatory responses (El-Maarri et al., 2011; Ghosh et al., 2017), no clear ART effect has been reported. Our results show that in term placentas for embryo culture groups, regardless the length of culture and sex, LINE elements are substantially hypomethylated compared to natural conceived groups, which might cause a dysregulation in placenta-specific gene expression.

### Reduced Fetal Growth and Further Loss of Placental DNA Methylation are Induced by Extended Embryo Culture

Superovulation using various gonadotropin dosages can result in changes in imprinted genes and fetal placental outcomes in mice (Sato et al., 2007; Fortier et al., 2008) (Market-Velker et al., 2010) (de Waal et al., 2012; Fortier et al., 2014) (Velker et al., 2017; Chen et al., 2018) (Yu et al., 2019; Huo et al., 2020; Vrooman et al., 2020). However, we recently showed that when comparing all ART procedures, including superovulation, *in vitro* fertilization, embryo culture, and embryo transfer, embryo culture led to the greatest impact on imprinted gene methylation, global DNA methylation as assessed by luminometric methylation assay, placental overgrowth, and reduction in fetal weight (Vrooman et al., 2020), therefore leading us to make it the focus of the present study.

Our study identified at least two critical windows of preimplantation development that adversely affect fetal and placental growth. Both 1-cell-morula and morula-blastocyst culture significantly reduce fetal/placental ratios. Intriguingly, the decrease in fetal/placental ratio in the 1-cell-morula group is associated with both a reduction in fetal weight and concomitant increase in placental weight, whereas the decrease in fetal/placental ratio in the morula-blastocyst group is only due to a reduction in fetal weight with no placental effects. These results likely suggest different mechanisms--culture after the morula stage affects fetal weight but does not appear to affect the placenta.

The results from this study suggest that the impacts of embryo culture on placenta are complex because the phenotypes associated with different culture periods coincide with different biological processes; placental morphological changes, loss of DNA methylation, and reduced fetal growth are independent effects. Furthermore, the phenotype from the one-cell to blastocyst stage appears to be a simple summation of these independent effects occurring during discrete windows of preimplantation development. Although the majority of the effects are linked to early preimplantation embryo development, the detectable differences in placental methylation and reduced fetal growth in the group cultured from morula-blastocyst stage suggest culture conditions should be optimized for this later window. Last, by identifying two windows of embryo culture that differentially and negatively impact fetal growth, our study highlights the importance of optimizing culture conditions for human preimplantation embryos to mitigate direct effects on the fetus as well as the secondary effect on fetal growth through placental defects. Further work is still needed to address the individual impacts of superovulation, embryo transfer, and other add-on assisted reproductive technologies on placentation and fetal development.

## Data Availability

The datasets presented in this study can be found in online repositories. The names of the repository/repositories and accession number(s) can be found below: https://www.ncbi.nlm.nih.gov/geo/, GSE193271.
