# Supplementary material for: Placental Abnormalities are Associated With Specific Windows of Embryo Culture in a Mouse Model
Source: Front Cell Dev Biol. 2022 Apr 25;10:884088. doi: 10.3389/fcell.2022.884088 (PMC9081528; doi:10.3389/fcell.2022.884088)
Supplement: Supplementary file 1 [file DataSheet1.docx]

**Supplemental Table 1. Litter characteristics of mouse concepti exposed to embryo culture during preimplantation development.**

|  | N* | Number of litters analyzed | Litter size | Implantation  Rate (%) | Live concepti rate (%) | Fraction male pups |
| --- | --- | --- | --- | --- | --- | --- |
| Natural** | 74 | 5 | 14.80 ± 1.07 | N/A*** | N/A*** | 0.50 |
| Morula control | 18 | 7 | 2.57 ± 0.43 | 32.86 ± 5.22 | 25.71 ± 4.29 | 0.50 |
| 4 cell-morula | 15 | 7 | 2.14 ± 0.55 | 31.41 ± 8.00 | 21.43 ± 5.53 | 0.60 |
| 1 cell-morula | 24 | 7 | 3.43 ± 0.87 | 44.29 ± 11.31 | 34.29 ± 8.69 | 0.29 |
| Blastocyst control | 22 | 6 | 3.67 ± 1.09 | 48.33 ± 11.95 | 36.67 ± 10.85 | 0.45 |
| Morula-blastocyst | 25 | 6 | 4.17 ± 0.95 | 55.00 ± 11.76 | 41.67 ± 9.46 | 0.68 |
| 1 cell-blastocyst | 20 | 5 | 4.00 ± 0.84 | 58.00 ± 12.41 | 40.00 ± 8.37 | 0.65 |

* Total number of concepti in each group.

** Natural group is shown for visual comparison but was not statistically tested.

*** Implantation and live concepti rate cannot be determined in Natural pregnancies since the number of preimplantation embryos in each pregnancy is unknown.

Litter sizes were compared by one-way ANOVA (p>0.05).

Implantation and live concepti rate were compared by a significant (p<0.05) global likelihood ratio test of differences between groups in the mixed effects model (p>0.05).

Fraction of male pups were compared by Fisher’s exact test (p>0.05).

**Supplemental Table 2. List of Differentially Methylated Regions located at or nearby imprinting control regions for both 1-cell-morula and 1-cell-blastocyst placentas versus naturally conceived placentas.**

| 1-cell-morula vs. Natural | 1-cell-blastocyst vs. Natural | Common ICRs |
| --- | --- | --- |
| *Ampd3* | *Ano1* | *Aqp1* |
| *Aqp1* | *Aqp1* | *Blcap* |
| *Blcap* | *Blcap* | *Calcr* |
| *Calcr* | *Calcr* | *Cdh15* |
| *Cd81* | *Cdh15* | *Commd1* |
| *Cdh15* | *Commd1* | *Dscam* |
| *Commd1* | *Ctnna3* | *Fkbp6* |
| *Dscam* | *Ddc* | *Gnas* |
| *Fkbp6* | *Dlk1* | *Grb10* |
| *Gnas* | *Dscam* | *H13* |
| *Grb10* | *Fkbp6* | *H19* |
| *H13* | *Fthl17a* | *Igf2r* |
| *H19* | *Gabra5* | *Impact* |
| *Igf2r* | *Gabrb3* | *Ins2* |
| *Impact* | *Gnas* | *Jade1* |
| *Ins2* | *Grb10* | *Kcnq1* |
| *Jade1* | *H13* | *Kcnq1ot1* |
| *Kcnq1* | *H19* | *Magi2* |
| *Kcnq1ot1* | *Igf2r* | *Mest* |
| *Magi2* | *Impact* | *Mirg* |
| *Mest* | *Ins2* | *Ndn* |
| *Mirg* | *Jade1* | *Nespas* |
| *Ndn* | *Kcnq1* | *Ntm* |
| *Nespas* | *Kcnq1ot1* | *Pde10a* |
| *Nnat* | *Lin28b* | *Peg10* |
| *Ntm* | *Magi2* | *Peg13* |
| *Pde10a* | *Mest* | *Peg3* |
| *Peg10* | *Mirg* | *Plagl1* |
| *Peg13* | *Mkrn3* | *Platr20* |
| *Peg3* | *Ndn* | *Rhox5* |
| *Plagl1* | *Nespas* | *Rian* |
| *Platr20* | *Ntm* | *Sgce* |
| *Rhox5* | *Pde10a* | *Slc22a3* |
| *Rian* | *Peg10* | *Slc38a4* |
| *Sgce* | *Peg13* | *Smoc2* |
| *Slc22a3* | *Peg3* | *Trappc9* |
| *Slc38a4* | *Plagl1* | *Trpm5* |
| *Smoc2* | *Platr20* | *Tspan32* |
| *Th* | *Pon2* | *Tssc4* |
| *Trappc9* | *Rhox5* | *Usp29* |
| *Trpm5* | *Rian* | *Zdbf2* |
| *Tspan32* | *Sgce* | *Zfp64* |
| *Tssc4* | *Slc22a3* | *Zrsr1* |
| *Usp29* | *Slc38a4* |  |
| *Zdbf2* | *Smoc1* |  |
| *Zfp64* | *Smoc2* |  |
| *Zrsr1* | *Thbs2* |  |
|  | *Trappc9* |  |
|  | *Trp73* |  |
|  | *Trpm5* |  |
|  | *Tspan32* |  |
|  | *Tssc4* |  |
|  | *Usp29* |  |
|  | *Zdbf2* |  |
|  | *Zfat* |  |
|  | *Zfp64* |  |
|  | *Zim3* |  |
|  | *Zrsr1* |  |

**Supplemental Table 3. Odds Ratio of DMR location in promoters and introns**

|  | Promoter Comparisons | *P*-value* | Ratio** |
| --- | --- | --- | --- |
| Sexes combined | 1-cell-Morula vs Natural | 9.9347x10^-150^ | 52X |
|  | 1-cell-Blastocyst vs Natural | 3.4440x10^-215^ | 55X |
| Females | 1-cell-Morula vs Natural | 4.064x10^-80^ | 76X |
|  | 1-cell-Blastocyst vs Natural | 3.2252x10^96^ | 63X |
| Males | 1-cell-Morula vs Natural | 1.2955x10^-62^ | 82X |
|  | 1-cell-Blastocyst vs Natural | 1.048x10^-108^ | 97X |
|  | **Intron Comparisons** | ***P*-value** | **Ratio** |
| Sexes combined | 1-cell-Morula vs Natural | 0.001645 | 1X |
|  | 1-cell-Blastocyst vs Natural | 5.2957x10^-09^ | 1X |
| Females | 1-cell-Morula vs Natural | 0.061511 | 1X |
|  | 1-cell-Blastocyst vs Natural | 0.061511 | 1X |
| Males | 1-cell-Morula vs Natural | 0.094545 | 1X |
|  | 1-cell-Blastocyst vs Natural | 0.0016644 | 1X |

*Groups were compared by Fisher’s exact test (p>0.05).

**Odds ratio that a DMR is more likely to be at promoters or introns than random regions by randomly picking the same number of regions anywhere in the genome.

**Supplemental Table 4. Odds Ratio of DMRs with repetitive elements is located in promoters and introns**

|  | Promoter Comparisons | *P*-value | Ratio |
| --- | --- | --- | --- |
| Sexes combined | 1-cell-Morula vs Natural | 0.00010 | 17X |
|  | 1-cell-Blastocyst vs Natural | 4.5171x10^-14^ | 33X |
| Females | 1-cell-Morula vs Natural | 5.8536x10^-05^ | 43X |
|  | 1-cell-Blastocyst vs Natural | 1.1161x10^-05^ | 23X |
| Males | 1-cell-Morula vs Natural | 1.4176x10^-10^ | 75X |
|  | 1-cell-Blastocyst vs Natural | 3.057x10^-12^ | 80X |
|  | **Intron Comparisons** | ***P*-value** | **Ratio** |
| Sexes combined | 1-cell-Morula vs Natural | 2.3364x10^-51^ | 6X |
|  | 1-cell-Blastocyst vs Natural | 7.0041x10^-63^ | 5X |
| Females | 1-cell-Morula vs Natural | 0.042376 | 2X |
|  | 1-cell-Blastocyst vs Natural | 2.8801x10^-05^ | 2X |
| Males | 1-cell-Morula vs Natural | 0.16957 | 1X |
|  | 1-cell-Blastocyst vs Natural | 0.077484 | 1X |

*Groups were compared by Fisher’s exact test (p>0.05).

**Odds ratio that a DMR is more likely to be at promoters or introns than random regions by randomly picking the same number of regions anywhere in the genome.

**Supplemental Table 5. E18.5 placental gene enrichment analysis using Bumphunter results for embryo culture groups compared to the naturally conceived group.**

| Gene | Group | Methylation status |
| --- | --- | --- |
| *Pramef12* | Tissue-Enriched | Hypo |
| *Adm* | Tissue-Enriched | Hypo |
| *Flt1* | Tissue-Enriched | Hypo |
| *Ghrh* | Tissue-Enriched | Hypo |
| *Tnfrsf9* | Tissue-Enriched | Hypo |
| *Foxo4* | Tissue-Enriched | Hypo |
| *Tex19.1* | Tissue-Enriched | Hypo |
| *Ctsj* | Tissue-Enriched | Hypo |
| *Peg10* | Tissue-Enriched | Hypo |
| *Pla2g4d* | Tissue-Enriched | Hypo |
| *Gjb3* | Tissue-Enriched | Hypo |
| *Sin3b* | Tissue-Enriched | Hypo |
| *Fthl17a* | Tissue-Enriched | Hypo |
| *Sbsn* | Tissue-Enriched | Hypo |
| *Pou2f3* | Tissue-Enriched | Hypo |
| *Nup62cl* | Tissue-Enriched | Hypo |
| *Plet1* | Tissue-Enriched | Hypo |
| *Slc22a3* | Tissue-Enriched | Hypo |
| *Hand1* | Tissue-Enriched | Hypo |
| *Nrk* | Tissue-Enriched | Hyper |
| *Csf1r* | Tissue-Enriched | Hypo |
| *Pcdh12* | Tissue-Enriched | Hyper |
| *Bhlha15* | Group-Enriched | Hypo |
| *Dtx1* | Group-Enriched | Hypo |
| *Tmc5* | Group-Enriched | Hypo |
| *Gata2* | Group-Enriched | Hyper |
| *Cdh5* | Group-Enriched | Hypo |
| *Atp6v0d2* | Group-Enriched | Hypo |
| *Slit1* | Group-Enriched | Hypo |
| *Zdbf2* | Group-Enriched | Hypo |
| *Slc30a2* | Group-Enriched | Hypo |
| *Fhdc1* | Group-Enriched | Hypo |
| *Msrb3* | Group-Enriched | Hypo |
| *Ppp1r15a* | Group-Enriched | Hypo |
| *Aqp1* | Group-Enriched | Hypo |
| *Anxa4* | Group-Enriched | Hypo |
| *Il2rg* | Group-Enriched | Hypo |
| *Slco2a1* | Group-Enriched | Hypo |
| *Il2rb* | Group-Enriched | Hypo |
| *Tead4* | Group-Enriched | Hypo |
| *Cand2* | Group-Enriched | Hyper |
| *Atp11a* | Group-Enriched | Hyper |
| *Morc4* | Group-Enriched | Hyper |
| *Rai14* | Group-Enriched | Hypo |
| *Loxl2* | Group-Enriched | Hypo |
| *Cma1* | Group-Enriched | Hypo |
| *Sdr42e1* | Group-Enriched | Hypo |
| *Myo1d* | Group-Enriched | Hypo |
| *Hrct1* | Group-Enriched | Hypo |
| *Myof* | Group-Enriched | Hypo |
| *Glis1* | Group-Enriched | Hypo |
| *Exoc3l4* | Group-Enriched | Hypo |
| *Sfrp4* | Group-Enriched | Hypo |
| *Krt25* | Group-Enriched | Hypo |
| *Aa467197* | Group-Enriched | Hypo |
| *Pde10a* | Group-Enriched | Hypo |
| *Epas1* | Group-Enriched | Hypo |
| *Krt8* | Group-Enriched | Hypo |
| *Maged2* | Group-Enriched | Hypo |
| *Kcnk5* | Group-Enriched | Hypo |
| *Slc16a9* | Group-Enriched | Hyper |
| *Krt7* | Group-Enriched | Hyper |
| *Fndc3b* | Tissue-Enhanced | Hypo |
| *Isg20* | Tissue-Enhanced | Hypo |
| *Pla2r1* | Tissue-Enhanced | Hyper |
| *Lama5* | Tissue-Enhanced | Hypo |
| *Peg3* | Tissue-Enhanced | Hypo |
| *Tceanc* | Tissue-Enhanced | Hyper |
| *Hspg2* | Tissue-Enhanced | Hypo |
| *Tram2* | Tissue-Enhanced | Hypo |
| *Zbtb7c* | Tissue-Enhanced | Hypo |
| *Gucy1b2* | Tissue-Enhanced | Hypo |
| *Arid3a* | Tissue-Enhanced | Hypo |
| *Dsg2* | Tissue-Enhanced | Hypo |
| *Dock5* | Tissue-Enhanced | Hypo |
| *Lmcd1* | Tissue-Enhanced | Hypo |
| *Fam83g* | Tissue-Enhanced | Hypo |
| *Gzmm* | Tissue-Enhanced | Hypo |
| *Col4a2* | Tissue-Enhanced | Hypo |
| *Afap1* | Tissue-Enhanced | Hypo |
| *Angpt2* | Tissue-Enhanced | Hypo |
| *Epop* | Tissue-Enhanced | Hypo |
| *Capg* | Tissue-Enhanced | Hypo |
| *Bmp8a* | Tissue-Enhanced | Hyper |
| *Ccl27a* | Tissue-Enhanced | Hypo |
| *Cldn4* | Tissue-Enhanced | Hypo |
| *Dnmt3l* | Tissue-Enhanced | Hypo |
| *Nostrin* | Tissue-Enhanced | Hypo |
| *Tfeb* | Tissue-Enhanced | Hypo |
| *Adgrf4* | Tissue-Enhanced | Hypo |
| *Slc38a4* | Tissue-Enhanced | Hypo |
| *Mbnl3* | Tissue-Enhanced | Hypo |
| *Acp7* | Tissue-Enhanced | Hypo |
| *Lgals9* | Tissue-Enhanced | Hypo |
| *Myom3* | Tissue-Enhanced | Hypo |
| *Slc16a3* | Tissue-Enhanced | Hypo |
| *Ppfibp2* | Tissue-Enhanced | Hypo |
| *Itga5* | Tissue-Enhanced | Hypo |
| *Muc15* | Tissue-Enhanced | Hypo |
| *Adamts2* | Tissue-Enhanced | Hypo |
| *Adam19* | Tissue-Enhanced | Hypo |
| *Slc38a1* | Tissue-Enhanced | Hypo |
|  |  |  |

**Supplemental Figure 1. Association between fetal weight and placental weight in individual E18.5 concepti with different embryo culture periods.** A) Scatter plot of fetal weight and placental weight for Natural (gray dots), Morula control (yellow dots), 4cell-morula (orange dots), and 1-cell-morula (red dots) culture groups. B) Scatter plot of fetal weight and placental weight for Natural (gray dots), Blastocyst control (green dots), Morula-blastocyst (blue dots), and 1-cell-blastocyst (purple dots) culture groups.


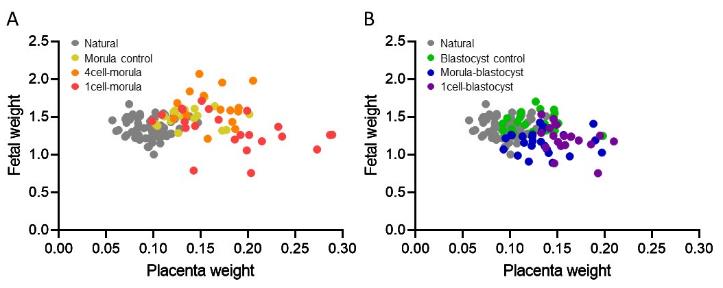


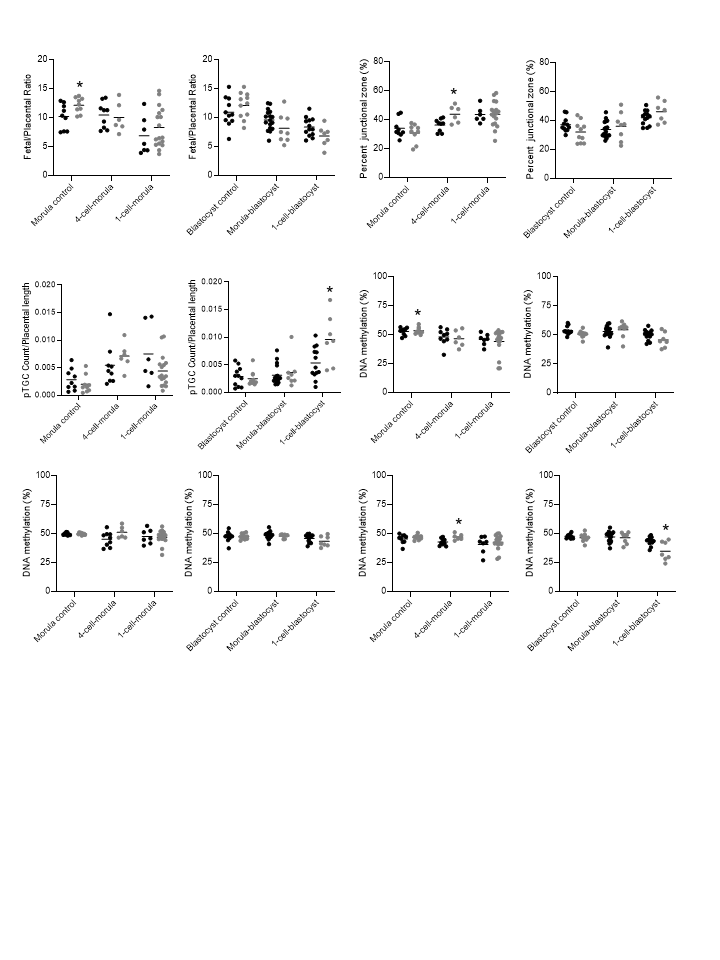


**Supplemental Figure 2. Comparison of males and females for fetal/placental ratio, junctional zone percentage, and ICR methylation levels for *H19*, *Kcnq1ot1*, and *Peg3* in morula and blastocyst transfer groups.** Black dots=male concepti, gray dots=female concepti. Asterisks denote significant differences between the sexes by Students t-test (p<0.05).

**Supplemental Figure 3. Distribution of repetitive elements included on the Infinium Mouse Methylation BeadChip array by class.** Each color represents a different class of repetitive element.


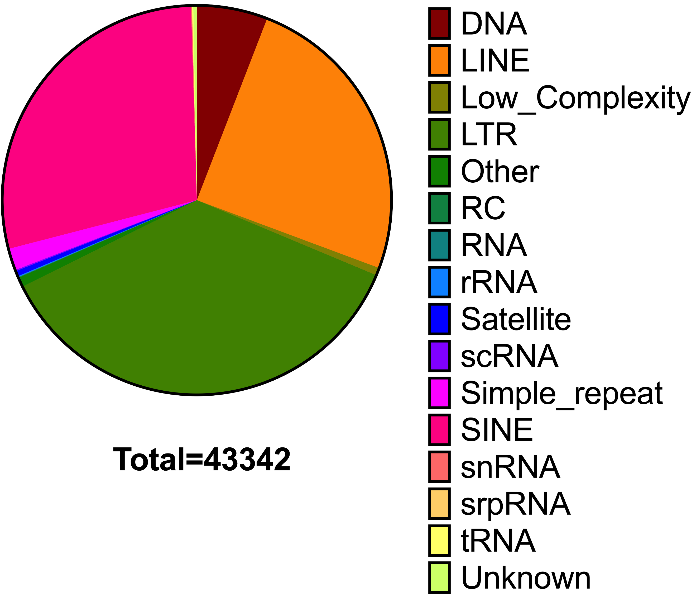


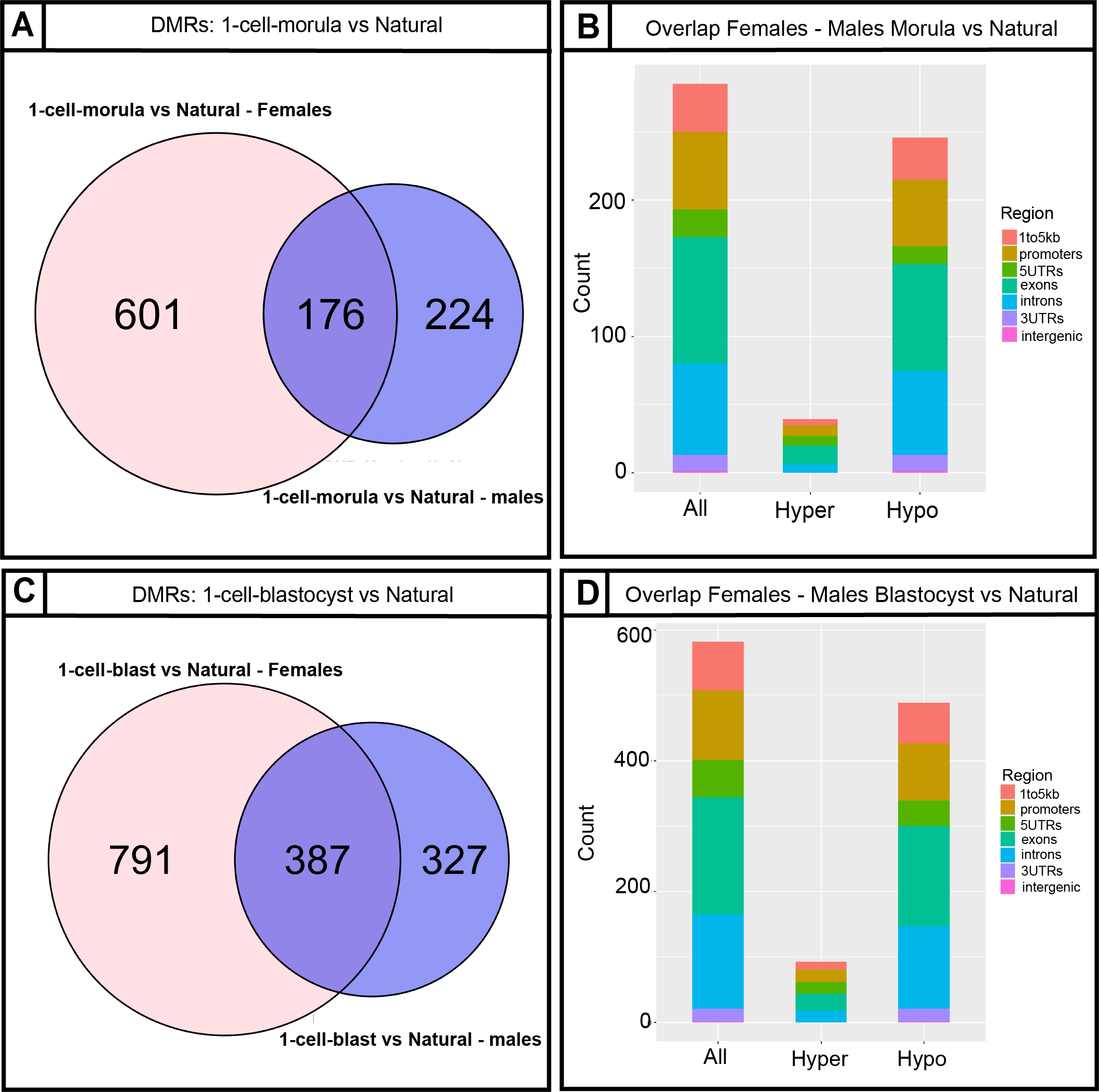


**Supplemental Figure 4. Sex-specific analyses of E18.5 placentas from 1-cell-morula and 1-cell-blastocyst compared to Natural controls.** Venn diagrams for Hypomethylated DMRs using package VennDiagram for R (this package considered multiple DMRs in a gene as one) to find overlap between two analyses: A) 1-cell-morula versus Natural females and 1-cell-morula versus Natural males and C) 1-cell-blastocyst versus Natural females and 1-cell-blastocyst versus Natural males. Bar graphs showing annotated overlap for all DMRs by predicted genomic regions using annotatr R package: B) 1-cell-morula versus Natural females and 1-cell-morula versus Natural males and D) 1-cell-blastocyst versus Natural females and 1-cell-blastocyst versus Natural males. Bar graphs were done using mm10/GRCm10 mouse genome assembly. The number of identified regions were higher than our number of DMRs because some DMRs overlap with more than one genomic locus.. For sex specific analyses, 1-cell-morula (n=6), 1-cell-blastocyst (n=6) and Natural were used for each sex. For Bumphunter analyses, cutoff value =0.1. For more details on the parameters used for Bumphunter, refer to Methods.

**Supplemental Figure 5.** Percent junctional zone comparison among samples used for genome-wide DNA methylation analyses. Males and females were compared within transfer groups by Student’s t-test. No significant differences were detected (p>0.05).


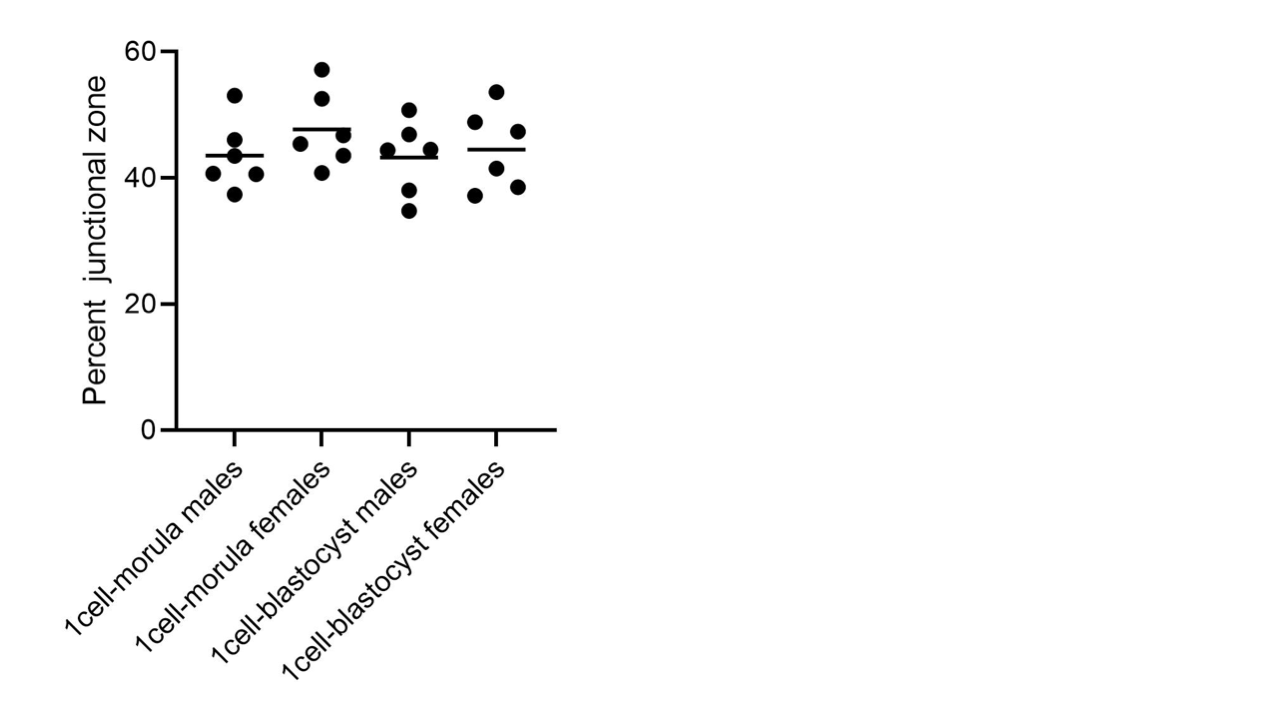


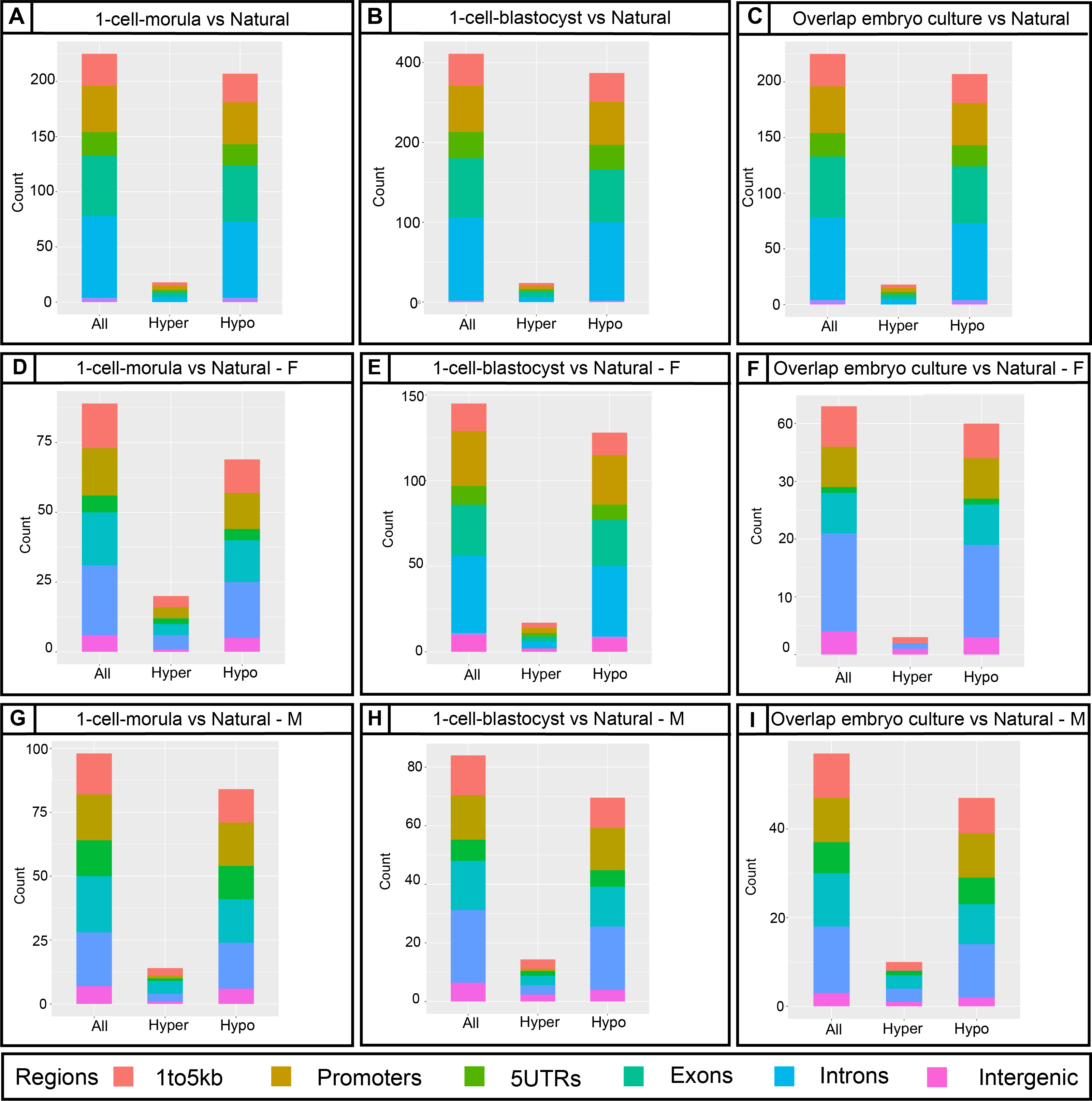


**Supplemental Figure 6. Genomic distribution of DMRs from the Bumphunter results using Repetitive Elements.** Bar graphs showing annotated DMRs containing repetitive elements by predicted genomic regions using annotatr R package: A, D, G) 1-cell-morula versus Natural for all, for females (F) and males (M). B, E, H) 1-cell-blastocyst versus Natural for all, for females (F) and males (M). C, F, I) 1-cell-morula versus Natural and 1-cell-blastocyst versus Natural for all, for females (F) and males (M). Bar graphs were done using mm10/GRCm10 mouse genome assembly, more DMRs are present in the graphs because some overlap with multiple genes and a single DMR could be present in more than one of the predicted regions. For all analyses, 1-cell-morula (n=12, 6 females and 6 males), 1-cell-blastocyst (n=12, 6 females and 6 males) and Natural (n=10, 5 females and 5 males). For Bumphunter analyses, cutoff value =0.1. For more details on the parameters used for Bumphunter, refer to Methods. Repetitive elements were located using the RepeatMasker tool and Genomic regions were found using the package annotr for R.

**
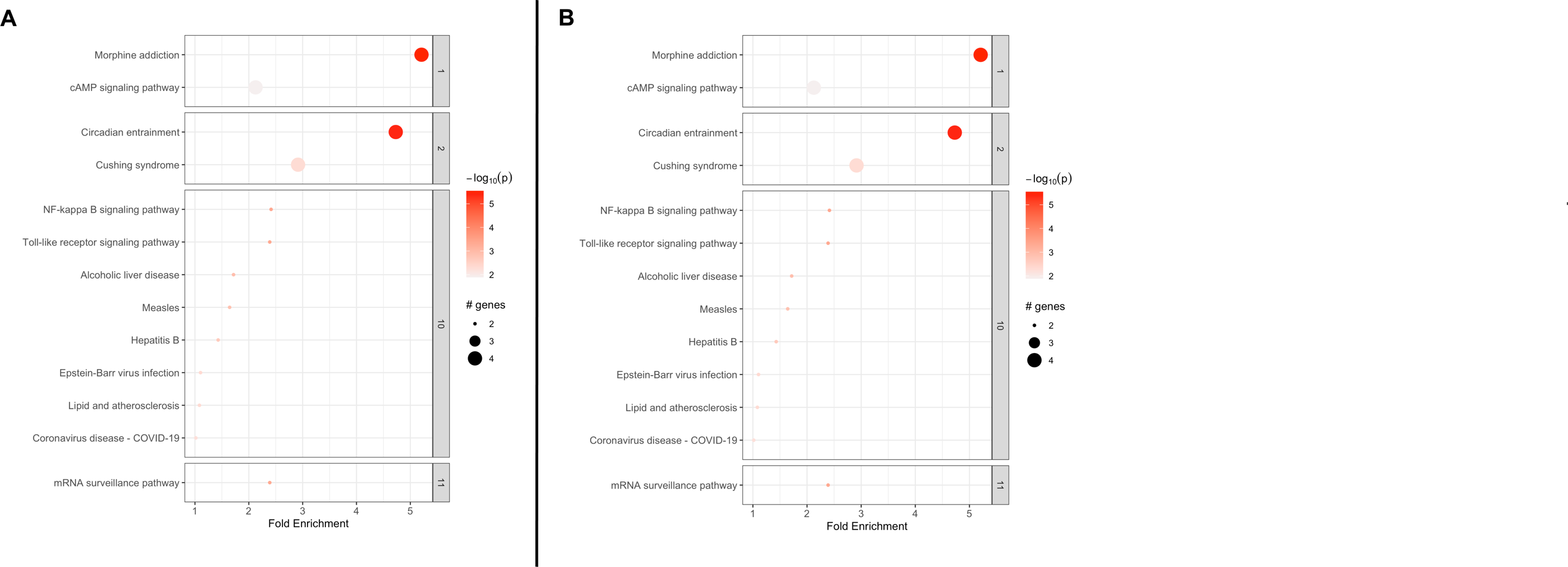
**

**Supplemental Figure 7. Gene ontology pathway analysis and placenta-specific gene analysis using DMRs affected by embryo culture**. Top gene ontology pathways affected by embryo culture using genes with affected DMRs at A) intronic regions and B) promoter regions, obtained by Bumphunter. The color indicates the P-value (dark orange=high, light orange=low). For more details on the parameters used for Bumphunter, refer to Methods.
